# *Staphylococcus aureus* hitchhiking from colonization to bacteremia via Candida within ICU infection prevention studies: a proof of concept modelling

**DOI:** 10.1007/s10096-023-04573-1

**Published:** 2023-03-06

**Authors:** James C. Hurley

**Affiliations:** 1 Melbourne Medical School, University of Melbourne, Melbourne, Australia; 2 Division of Internal Medicine, Grampians Health Ballarat, PO Box 577, Ballarat, VIC 3353 Australia

**Keywords:** *Staphylococcus aureus*, Bacteremia, Candidemia, Structural equation modelling

## Abstract

Whether *Candida* within the patient microbiome drives the pathogenesis of *Staphylococcus aureus* bacteremia, described as microbial hitchhiking, cannot be directly studied. Group-level observations from studies of various decontamination and non-decontamination-based ICU infection prevention interventions and studies without study interventions (observational groups) collectively enable tests of this interaction within causal models. Candidate models of the propensity for *Staphylococcus aureus* bacteremia to arise with versus without various antibiotic, anti-septic, and antifungal exposures, each identified as singleton exposures, were tested using generalized structural equation modelling (GSEM) techniques with *Candida* and *Staphylococcus aureus* colonization appearing as latent variables within the models. Each model was tested by confrontation against blood and respiratory isolate data, obtained from 467 groups within 284 infection prevention studies. Introducing an interaction term between *Candida* colonization and *Staphylococcus aureus* colonization substantially improved GSEM model fit. Model-derived coefficients for singular exposure to anti-septic agents (− 1.28; 95% confidence interval; − 2.05 to − 0.5), amphotericin (− 1.49; − 2.3 to − 0.67), and topical antibiotic prophylaxis (TAP; + 0.93; + 0.15 to + 1.71) as direct effects versus *Candida* colonization were similar in magnitude but contrary in direction. By contrast, the coefficients for singleton exposure to TAP, as with anti-septic agents, versus *Staphylococcus* colonization were weaker or non-significant. Topical amphotericin would be predicted to halve both candidemia and *Staphylococcus aureus* bacteremia incidences versus literature derived benchmarks for absolute differences of < 1 percentage point. Using ICU infection prevention data, GSEM modelling validates the postulated interaction between Candida and *Staphylococcus* colonization facilitating bacteremia.

**Supplementary Information:**

The online version contains supplementary material available at 10.1007/s10096-023-04573-1.

## Introduction

Numerous animal models implicate candida colonization facilitating invasive bacterial infections, a process that has been described as “microbial hitchhiking” [1–4]. Reconciling the extensive pre-clinical evidence base implicating “microbial hitchhiking” versus the paucity of clinical evidence for this interaction within individual patients remains challenging [5–7]. On the one hand, measuring the *Staphylococcal* bacteremia incidence among individual patients receiving interventions to alter candida colonization would be logistically complex for multiple reasons. Blood stream infection (BSI) endpoints are generally uncommon or rare, the key body site location of any postulated interaction, whether the oropharynx or elsewhere, remains unclear, and measuring colonization, whether bacterial or candida, is problematic. Moreover, the specific mechanisms mediating the “microbial hitchhiking,” whether the detectable presence versus the functional activity of candida colonization, remains uncertain.

On the other hand, the numerous studies of various interventions for preventing infection acquired by patients receiving mechanical ventilation (MV) within the intensive care unit (ICU) literature can be perceived “in toto” as a single natural experiment of the group level effects of a range of antibiotic, anti-fungal and anti-septic exposures given as prophylaxis. These exposures are known to influence colonization with pathogenic bacteria and Candida [8]. Of note, the antibiotic-based interventions, as selective digestive decontamination (SDD) and selective oropharyngeal decontamination (SOD), combine two singleton exposures, being topical antibiotic prophylaxis (TAP) and antifungal prophylaxis. Moreover, from the first SDD/SOD study [9], TAP use within the ICU context was presumed to induce contextual effects mediated via the ICU microbiome with potential to spill over into concurrent control group patients. Several SDD studies deliberately avoided these contextual effects by using either non-concurrent or no control group patients in the study [10–13].

**Fig. 1:**
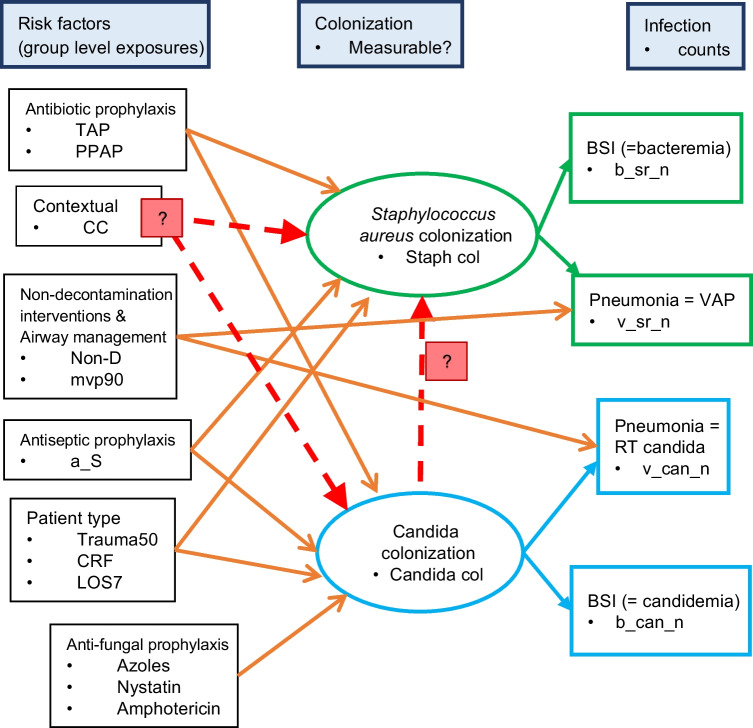
Causal model of factors bearing on the interaction between *Staphylococcus aureus* and Candida colonization towards causing blood stream and other infections. The dotted red arrows and red boxes labelled “?” label the elements required to address the two research questions here. Within each element in the figure are the abbreviations that correspond to factors in the GSEM models, “contextual” refers to the contextual effect for concurrent control (CC) groups concurrent to use of antibiotic-based interventions (TAP) in the ICU, CRF is patient selection for candidemia risk factors; BSI is blood stream infection; non-D is non-decontamination intervention; MVP90 is use of mechanical ventilation by more than 90% of the group for > 24 h; trauma50 are an ICU with more than 50% of admissions for trauma and LOS7 is a mean or median length of ICU stay for the group of more than 7 days

The postulated “hitchhiking” and contextual exposures within the ICU environment as facilitators of *Staphylococcal* bacteremia could be posed as research questions versus other drivers of bacteremia within a causal model (Fig. 1) as has recently been demonstrated in the case of Candida facilitating Pseudomonas bacteremia [14]. Structural equation modelling (SEM) is an emerging method to test for potential causal relationships between multiple simultaneously observed variables mediated through latent variables [15–17]. SEM is used here to test candidate models of interaction and various ecological effects by confrontation with the collective observations from published studies of ICU infection prevention interventions.

## Materials and methods

There are three objectives here. Firstly, to recapitulate the indicative effect size for the various infection prevention interventions versus end points of Candida and *Staphylococcal* infection within the literature using meta-analysis. Second, to test for both the postulated interaction between Candida and *Staphylococcal* colonization, and for the contextual effect of concurrency to TAP use, each within a postulated causal model of bacteremia pathogenesis (Fig. 1). These models are developed through confrontation of candidate models with group level infection and exposure data using generalized structural equation modelling (GSEM) methods. The third objective is to estimate the relative impacts of exposures to the use of TAP, anti-septic, antibiotic, and specific anti-fungal agents as singleton or compound exposures on bacteremia and candidemia versus literature derived benchmarks within the optimal GSEM model. Being an analysis of published work, ethics committee review of this study was not required.

### Study selection and decant of groups

The literature search and study decant used here is as described previously [14] and is detailed in electronic supplementary material (ESM) Fig S1. The key inclusion criterion, being patient groups requiring prolonged (> 24 h) ICU stay with the majority receiving MV within studies of ICU infection prevention interventions, was expanded with the requirement that the included studies have group level *Candida* or *Staphylococcus aureus* infection incidence proportion data. The studies were streamed into four broad categories of infection prevention intervention, being non-decontamination based, anti-septic based, antibiotic-based or single anti-fungal (SAF) based methods. Note that the antibiotic-based methods originate predominantly from studies of SDD/SOD regimens with multiple and various combinations of different antibiotic and anti-fungal components [18–26].

Studies without ICU infection prevention interventions (observational studies) were sourced to provide incidence proportion data from which to derive external benchmarks. Most of the studies had been cited in systematic reviews with additional studies being found by snowball sampling using the “Related articles” function within Google Scholar.

### Data extraction

The incidence proportions of VAP and BSI, however defined in each study, in association with *Candida* and *Staphylococcus aureus* were extracted. As *Candida* is generally not considered a cause of VAP, the count of *Candida* as a respiratory tract (RT *Candida*) isolate among patients with suspected VAP was recorded. These counts were each transformed to proportions using the number of patients with prolonged (> 24 h) ICU stay as the denominator.

### Indicative intervention effect sizes

The effect of each broad category of intervention versus the incidence proportions of VAP and BSI in association with *Candida* and *Staphylococcus aureus* were estimated using random effect meta-analysis. The effect sizes here are merely indicative as the various infection prevention interventions studied variously included both singleton and compound interventions. Moreover, they do not distinguish the contextual effects arising from intervention exposure on control groups concurrent within the same ICU versus non- concurrent.

### GSEM model components

The incidence proportions of VAP and BSI in association with *Candida* and *Staphylococcus aureus* are the measurement components. The following, each being group wide exposures, constitute the indicator variables of the GSEM models; origin from trauma ICU’s, being defined here as an ICU with > 50% of admissions being for trauma, whether more than 90% of patients of the group received more than 24 h of MV, and a mean (or median) length of ICU stay (ICU-LOS) for the group greater than 7 days. In the extraction of MV percentages, if this was not stated for any group, a percentage of less than 90% was assumed. A binary variable for ICU-LOS being greater or less than 7 days was derived with the mean (or median) length of mechanical ventilation was used as surrogate measures if the length of ICU-LOS was not available.

Also, the group wide presence of candidemia risk factors (CRF), such as liver transplantation or liver failure, use of parenteral nutrition, surgery for intestinal perforation, pancreatitis, and being colonized with *Candida*, however that was defined, as a basis for patient selection, were noted. Anti-septic interventions include chlorhexidine, povidone-iodine and iseganan regardless of whether the application was to the oropharynx, by tooth-brushing or by body-wash used as prophylaxis.

Antibiotic-based interventions typically combine TAP with an anti-fungal, together with or without Protocolized parenteral antibiotic prophylaxis (PPAP) [8]. TAP generally comprises non-absorbable antibiotics, such as polymyxin and various aminoglycosides, applied to either or both the oropharynx and gastrointestinal tract. The PPAP is the prophylactic use of a parenteral antibiotic, most commonly cefotaxime, as dictated by the study protocol whether to the intervention group alone or to both control and intervention groups (duplex studies).

Exposure to anti-fungal prophylaxis was identified whether as single anti-fungal agents (SAF) or as a combination intervention together with TAP exposures as within SDD or SOD regimens. These anti-fungal exposures were classified in line with [27, 28] into three categories; topical amphotericin, topical nystatin or an absorbable agent such as an azole anti-fungal used as prophylaxis.

### Structural equation modelling

Generalized structural equation modelling (GSEM) methods are an extension of SEM methods applied to count data. In the GSEM models, the VAP and BSI incidence proportion data, serve as the measurement components, the group level exposure parameters serve as the indicator variables and each of *Candida* colonization and *Staphylococcus aureus* colonization, being represented as latent variables, link the indicator and measurement components.

Three candidate GSEM models, corresponding to the research questions posed in Fig. 1, were tested. The first two (Model B and C), with and without the inclusion of an interaction terms between the latent variables, being *Candida* colonization and *Staphylococcus aureus* colonization. The third GSEM model (Model A) additionally includes an indicator term for concurrent control group membership within an antibiotic-based study to identify postulated contextual effects from TAP used concurrently within the ICU.

Study identifiers were used in the models to enable generation of robust variance covariance matrices of the coefficient estimate parameters of observations clustered by study. The GSEM model with the lowest Akaike's information criterion (AIC) score was selected as having parsimony and optimal fit from among the candidate models using the “GSEM” command in Stata (Stata 17, College Station Texas, USA) [29]. The post-model predictions were obtained using the command “nlcom” to obtain nonlinear combinations of estimators.

## Data availability of data and materials

All data generated or analyzed during this study are included in this published article and the ESM.

## Results

### Characteristics of the studies

Of the 288 studies identified by the search, 157 were sourced from 23 systematic reviews (Table 1; Fig S1; Table S1 – S5). Most studies were published between 1990 and 2010 and most had a mean ICU-LOS exceeding seven days. Twelve studies had more than one type of intervention group and 14 studies had either more than one or no control group. Most groups from observational studies had more than 150 patients per group versus less than 150 patients in the groups of the interventional studies.

**Table 1:** Characteristics of studies

	Observational	Non- decontamination	Topical anti-septic ^a^	Antibiotic based ^b^	Single anti-fungal ^c^
Study characteristics					
Listing	Table S1	Table S2	Table S3	Table S4	Table S5
Number of studies (*n*) ^d^	144	45	18	70	13
MV for >24 hours for < 90% (*n*)^e^	43	0	8	17	6
PPAP for control groups (*n*)	0	0	0	8	0
Trauma ICUs (*n*) ^f^	26	9	3	11	0
CRF as selection criteria (*n*) ^g^	10	0	0	8	6
Paediatric ICU (*n*)			1	1	
North American ICU (*n*)	34	9	8	6	2
Study publication year (range)	1987−2020	1987−2017	2000−2018	1984−2022	1994−2014
Group characteristics					
Number of groups (*n*) ^d^	163	90	39	142	33
Numbers of patients per study group; median (IQR) ^h^	280 (118−674)	75 (61−147)	130 (72−361)	48 (31−80)	69 (49−75)
Mean Length of stay < 7 days; (*n*) ^i^	28	14	14	14	2
Indicative intervention effect size (VAP / RT candida) ^j, k^					
VAP *Staphylococcus aureus* prevention effect (odds ratio; 95% CI; *n*)	NA	0.78; 0.66−0.93 (43)	0.53; 0.37−0.76 (12)	0.54; 0.42−0.68 (44)	NR
RT candida prevention effect (odds ratio; 95% CI; *n*)	NA	0.63; 0.43−0.93 (18)	0.24; 0.07−0.79 (7)	0.8; 0.41−1.57 (19)	NR
Indicative intervention effect size ^j, l^ (Bacteremia/Candidemia)					
*Staphylococcus aureus* bacteremia prevention effect (figure s2) (odds ratio; 95% CI; *n*)	NA	NR	1.01 0.74−1.37 (10)	0.98; 0.69−1.38 (25)	NR
Candidemia prevention effect (figure s3) (odds ratio; 95% CI; *n*)	NA	NR	0.75 0.55−1.03 (7)	0.52; 0.31−0.87 (23)	0.33; 0.15−0.74 (9) ^m^

*MV* mechanical ventilation; *PPAP* protocolized parenteral antibiotic prophylaxis; *NA* not applicable; *NR* not reported; *ICU* intensive care unit; *CI* confidence interval

^a^Among anti-septic studies, topical chlorhexidine was used in 15 intervention groups

^b^Among TAP intervention groups, the most common antibiotic combination used were polymyxin in combination with an aminoglycoside in 63 groups. Also, a topical anti-fungal was used in all but eight interventions groups, with amphotericin being the most common anti-fungal (50 intervention groups)

^c^Fluconazole was the most common single agent antifungal, used in seven intervention groups

^d^Note, several studies had more than one control and or intervention group. Hence the number of groups does not equal the number of studies

^e^Number of studies for which less than 90% of patients were reported to receive > 24 h of MV. MV proportion data was missing for 38 groups

^f^Number of trauma ICU’s; trauma ICU arbitrarily defined as an ICU with more than 50% of admissions for trauma

^g^Number of studies for which the presence of Candidemia risk factors (CRF) was used as study inclusion criteria

^h^Data is median and inter-quartile range (IQR)

^i^Number of studies for which the mean length of stay was less than 7 days. LOS data was missing for 25 groups

^j^Note that studies with zero events in both control and intervention arms do not contribute in the calculation of summary effects size. Summary effect sizes were derived using the Peto’s log odds ratio. Effect sizes not shown where derived from fewer than three studies

^k^Effect size is indicative for each category. Anti-septic interventions include Iseganin in one study; TAP interventions were usually in combinations with an anti-fungal agent

^l^Effect size is indicative as several interventions with combinations of agents have been included. TAP interventions were usually in combinations with an anti-fungal agent; SAF interventions were either nystatin (six intervention groups) or azole anti-fungal agents (nine intervention groups)

^m^Summary effect size from seven SAF studies that used nystatin was 1.2 (0.79–1.83) and from 9 studies that used an azole was 0.21 (0.11 – 0.4)

### VAP, bacteremia, and candidemia incidences

**Fig. 2:**
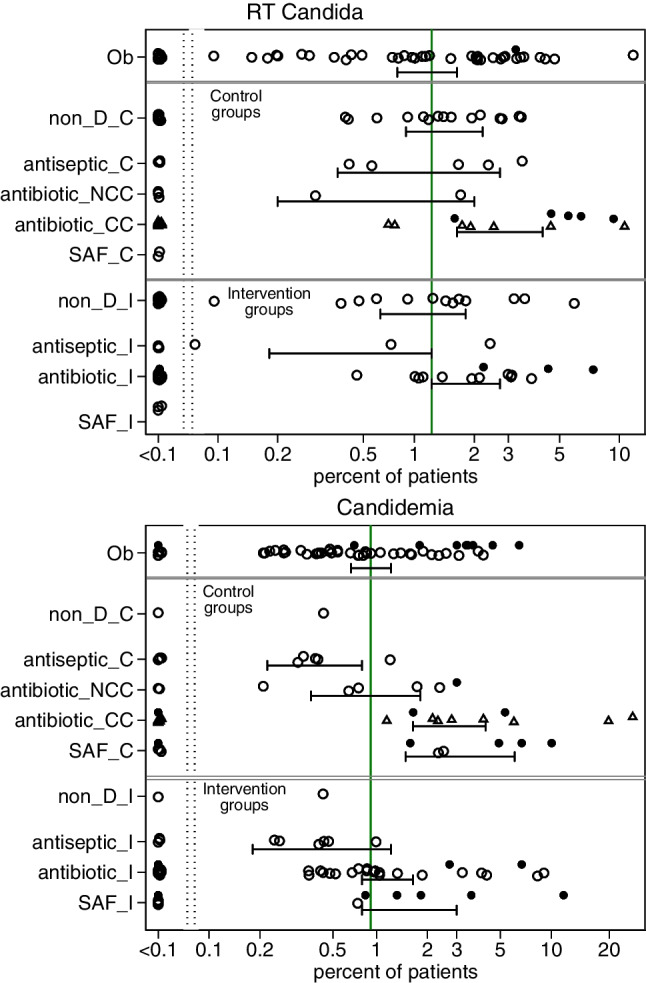
**(a & b)** Scatter plots, on a logit scale, of the incidence proportions of RT *Candida* (Fig. 2a) and candidemia (Fig. 2b) for groups from 288 studies as listed in tables S1 to S5. The mean proportion (and 95% CI) derived by random effect meta-analysis for each category of component (observational [Ob], control [_C] and intervention [_I]) group derived from observational [Ob], non-decontamination (non-D), antiseptic based, antibiotic-based and single anti-fungal (SAF) studies, is displayed. In each plot, the benchmark proportion (solid vertical line) is the mean proportion derived from the observational groups. Those component groups that did (●) versus did not (○) select patients with Candidemia risk factors (CRF) are indicated. All groups shown as (open circles) with the exception of concurrent control groups antibiotic-based interventions (CC is concurrent control; triangles), NCC is non-concurrent control (open circles)

**Fig. 3:**
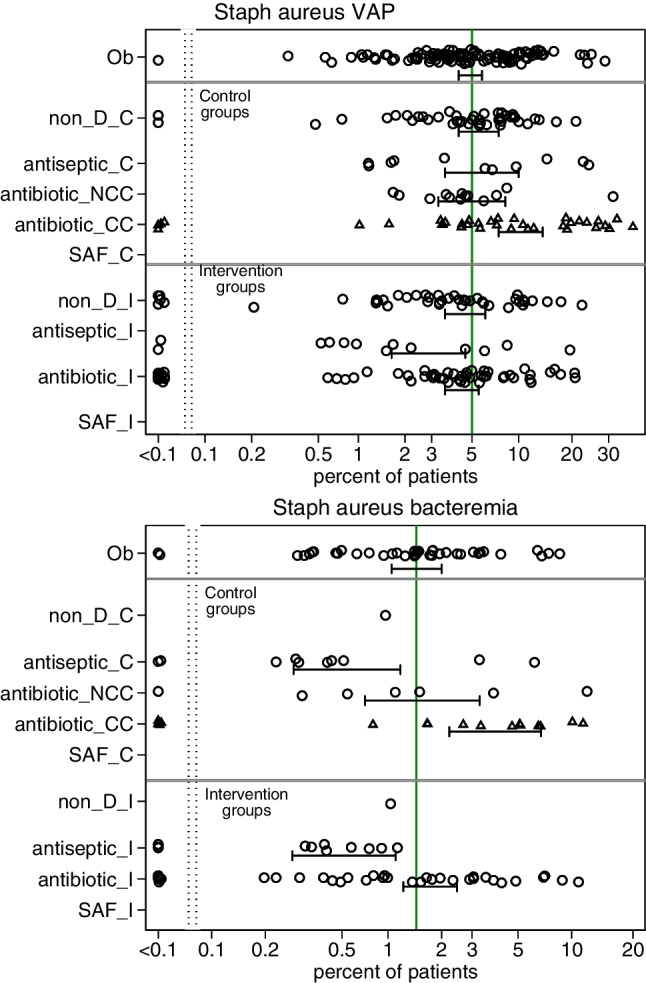
**(a & b)** Scatter plots, on a logit scale, of the incidence proportions of *Staphylococcus* VAP (Fig. 3a) and *Staphylococcus* bacteremia (Fig. 3b) for groups from 288 studies as listed in tables S1 to S5. The mean proportion (and 95% CI) derived by random effect meta-analysis for each category of component (observational [Ob], control [_C] and intervention [_I]) group derived from observational [Ob], non-decontamination (non-D), antiseptic based, antibiotic-based and single anti-fungal (SAF) studies, is displayed. In each plot, the benchmark proportion (solid vertical line) is the mean proportion derived from the observational groups. All groups shown as (open circles) with the exception of concurrent control groups antibiotic-based interventions (CC is concurrent control; triangles), NCC is non-concurrent control (open circles)

The incidence proportions of BSI and VAP with each of *Candida*, *Staphylococcus aureus* ranged approximately 100-fold across the various observational, control and intervention groups of the 288 studies (Figs. 2–3). These proportions were generally higher among studies of antibiotic-based interventions, particularly so for the concurrent control groups of these studies, versus a benchmark derived from observational groups. The candidemia incidence proportion was higher among groups from SAF studies as patient inclusion was often limited to those with CRF.

### Indicative effect size

The indicative prevention effects for three categories of interventions versus *Staphylococcus aureus* VAP were apparent for the anti-septic and the combination antibiotic-antifungal-based interventions. However, no category of intervention showed prevention effects versus *Staphylococcus aureus* bacteremia (Table 1). In the prevention of candidemia, the summary effects for the SAF and the combination antibiotic-antifungal-based interventions (TAP), except for those including nystatin which were without effect, were similar (Table 1; Fig. S3).

### GSEM modelling

**Fig. 4:**
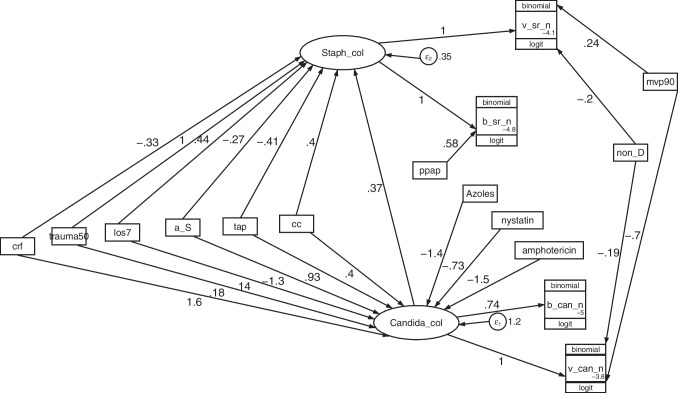
GSEM of the interaction model (Model A) in relation to *Staphylococcus* and *Candida* infection data. *Candida* col and *Staphylococcus* col (ovals) are latent variables representing *Candida* and *Staphylococcus* colonization, respectively. The variables in rectangles are binary predictor variables representing the group level exposure to the following; patient selection for candidemia risk factors (CRF); trauma ICU setting (trauma50), mean or median length of ICU stay ≥ 7 days (los7), exposure to a topical anti-septic (a_S), exposure to TAP (tap), concurrency of a control group with an antibiotic-based intervention group (CC), exposure to a non-decontamination based prevention method (non-D), greater than 90% use of mechanical ventilation (mvp90) or exposure to PPAP (ppap). Note that the model factorizes exposures from compound regimens (e.g., SDD and SOD, which combine TAP, an antifungal together with or without PPAP) into singleton TAP, PPAP and anti-fungal exposures. The circles contain error terms. The three part boxes represent the binomial proportion data for *Candida* and *Staphylococcus* VAP (v_can_n, v_sr_n) and candidemia (b_can_n) or bacteremia (b_sr_n) counts with the number of patients as the denominator which is logit transformed using the logit link function in the generalized model

The introduction of firstly an interaction term between *Candida* colonization and *Staphylococcus aureus* colonization (model C to model B) (Figs. S4 & S5), and then, the contextual effect of control group concurrency within a study of a TAP-based interventions as an indicator variable (model B to model A) (Figs. S5 & Fig. 4, sequentially improved the model fit as reflected in the AIC score towards the optimal model (model A) (Table 2; Fig. 4).

**Table 2:** Development of GSEM models; model C, model B & model A ^a^^, b^

	*Model C*	*Model B*	*Model A*	
	Fig S4	Fig S5	Figure 4	
				95%CI (Model A)
Factor (label abbreviations as in the models) ^c − k^				
b_Sr_n				
Staphylococcal colonization	1.01***	1.0***	1.0***	0.77 to 1.23
Ppap	0.63	0.6	0.58	− 0.06 to 1.22
_ Constant	− 4.84***	− 4.76***	− 4.79***	− 5.05 to -4.5
v_Sr_n				
Staphylococcal colonization	1	1	1	(constrained)
mvp90	0.3	0.23	0.24	− 0.18 to 0.66
non_D	− 0.27	− 0.27	− 0.2	− 0.48 to 0.07
_ Constant	− 4.21***	− 4.03***	− 4.09***	− 4.8 to -3.3
Staphylococcal colonization ^i^				
CC (Concurrency to TAP use)			0.4*	0.02 to 0.72
Tap	− 0.54***	− 0.47**	− 0.41*	− 0.7 to − 0.12
Anti-septic	− 0.76***	− 0.3	− 0.27	− 0.68 to 0.13
Los7	0.57***	0.46*	0.44**	0.15 to 0.73
Trauma 50	1.10***	1.05***	1.03***	0.72 to 1.3
Crf	0.36	− 0.19	− 0.33	− 0.8 to 0.14
Candida colonization		0.38***	0.37***	0.25 to 0.49
b_can_n				
Candida colonization	0.73***	0.73***	0.74***	0.38 to 1.1
_ Constant	− 5.05***	− 5.02***	− 5.04***	− 5.4 to − 4.7
v_can_n				
Candida colonization	1	1	1	(constrained)
mvp90	− 0.85	− 0.71	− 0.7	− 1.5 to 0.09
non_D	− 0.2	− 0.24	− 0.19	− 0.75 to 0.38
_ Constant	− 3.51***	− 3.76***	− 3.82***	− 5.4 to − 2.27
Candida colonization^j^				
CC (Concurrency to TAP use)			0.4	− 0.3 to 1.1
Tap	0.79	0.87*	0.93*	0.3 to 1.7
Anti-septic	− 1.38**	− 1.33**	− 1.28**	− 2.1 to − 0.49
Los7	0.12	0.16	0.14	− 0.4 to 0.67
trauma 50	0.17	0.19	0.18	− 0.65 to 0.99
Crf	1.55**	1.59**	1.56**	0.59 to 2.5
Amphotericin	− 1.55**	− 1.59***	− 1.56***	− 2.3 to − 0.65
Nystatin	− 0.43	− 0.76	− 0.73	− 1.9 to 0.43
Azoles	− 1.44**	− 1.5**	− 1.44**	− 2.5 to − 0.41
Error terms				
var (e. Staph col)	0.54***	0.37***	0.35***	0.26 to 0.49
var (e. Candida col)	1.31***	1.2***	1.18***	0.79 to 1.8
Model fit^k^				
AIC	4276	4234	4225	-
Groups (n)	473	473	473	-
Clusters (n)	288	288	288	
Factors	29	30	32	-

^a^Legend: *p < 0.05; **p < 0.01; ***p < 0.001

^b^Shown in this table are models derived with all studies derived as indicated in the figures corresponding to model C (Figure S4), model B (Figure S5) and model A (Fig. 4)

^c^v_sr_n is the count of *Staphylococcoal* VAP; v_can_n is the count of *RT Candida*; b_sr_n is the count of *Staphylococcoal* bacteremia; and b_can_n is the count of Candidemia; Staph col is Staphylococcal colonization; Candida col is Candida colonization

^d^PPAP is the group wide use of protocolized parenteral antibiotic prophylaxis; tap is topical antibiotic prophylaxis; non-D is a non-decontamination intervention

^e^MVP90 is use of mechanical ventilation by more than 90% of the group for > 24 h

^f^Crf is group wide exposure to a candidemia risk factor

^g^LOS7 is a mean or median length of ICU stay for the group of more than 7 days

^h^Trauma 50 is an ICU for which > 50% of admissions were for trauma

^i^*Staphylococcoal* colonization (*Staphylococcoal* col) is a latent variable

^j^*Candida* colonization (*Candida* col) is a latent variable

^k^Model fit; AIC is Akaike’s information criteria. This indicates model fit taking into account the statistical goodness of fit and the number of parameters in the model. Lower values of AIC indicate a better model fit. Groups is the number of patient groups; clusters is the number of studies; factors is the number of parameters in the model

In the optimal model (model A; Table 2; Fig. 4), the coefficients for singleton exposure to anti-septic agents (− 1.27; − 2.05 to − 0.5), amphotericin (− 1.49 -2.3 to − 0.66), patient selection for CRF (+ 1.55; 0.59 to 2.51) and TAP (+ 0.93; + 0.15 to + 1.71) versus *Candida* colonization were all similar in magnitude but contrary in direction. By contrast, among the group wide exposures versus *Staphylococcus aureus* colonization, these were generally weaker, less consistent between models and variably significant, with the exception of origin from a trauma ICU, which showed a consistently strong and positive association.

In all models, group wide exposure to CRF, anti-septics and singular exposures to each of TAP and antifungals, with the exception of nystatin, displayed strong and significant associations with the Candida colonization latent variable, and these were generally consistent across all three models.

The size of the positive effect of the interaction between Candida colonization on *Staphylococcus aureus* colonization was similar to the size of the positive effect of concurrency within an ICU studying an antibiotic-based interventions.

### Post-GSEM modelling predictions

Post model predictions of *Staphylococcus aureus* bacteremia (Fig S6a) and Candidemia (Fig S6b) incidence proportions were estimated for a putative group of non-trauma ICU patients with group mean LOS greater than seven days and without patient selection for CRF. Predictions were made for various combination and group wide singleton exposures to anti-septic agents, TAP, PPAP, nystatin, and amphotericin versus respective benchmarks derived for an equivalent putative non-concurrent control group. In every case, singleton exposure to either the anti-fungal amphotericin or to anti-septics outperformed singleton exposure to TAP towards lower predicted bacteremia incidence. Exposure to TAP combined with amphotericin, but not nystatin, was associated with significantly lower predicted *Staphylococcus aureus* bacteremia incidence versus benchmark. Similar differences versus benchmark were noted with respect to Candidemia predictions.

On the other hand, a significantly higher *Staphylococcus aureus* bacteremia incidence versus benchmark was projected in association with membership of a concurrent control group within an ICU exposed to an antibiotic-based intervention. Of note, the absolute differences, noted above, versus each benchmark are in each case no greater than approximately one percentage point.

## Discussion

Candida colonization of ICU patients is associated with poor patient outcomes including higher ICU mortality. This association is disproportionate to the scarcity of invasive candida infections among this patient population. Randomized controlled trials evaluating anti-fungal prophylaxis among ICU patients are difficult to undertake and the results for any end point are few and inconclusive [4–7].

The postulate, that interaction between Candida and *Staphylococcal* colonization facilitates bacteremia occurrence, is supported by extensive preclinical evidence but proof of concept in the clinical context is lacking [1–8]. Here, the “hitchhiking” postulate, and the postulated effect of concurrency, have been tested by confronting three candidate causal models with published data from broadly selected ICU infection prevention studies wherein groups of patients had received various study interventions and other exposures. The optimal GSEM model (Model A) includes both the postulated interaction between Candida and *Staphylococal* colonization together with the contextual influence of concurrency to TAP use within the ICU.

In confronting Model A with published group level infection data, three expected findings emerge. Trauma ICU admission is a risk factor for *Staphylococcus aureus* colonization, anti-fungal agents, such as azoles and amphotericin, and anti-septic agents, each showed strong prevention effects versus Candida colonization, whereas TAP as a singleton exposure showed strong promotion of Candida colonization. Two unexpected findings are that each of TAP and anti-septic agents show weak prevention versus *Staphylococcus aureus* colonization. Moreover, the strength of the “hitchhiking” and concurrency effects on *Staphylococcal* colonization are similar in size and direction within the optimal GSEM model (Model A).

The indicative effects sizes derived here (Table 1) are similar to summary effect sizes reported elsewhere in the literature for these interventions (ESM Table S6) [18–26]. Of note, these indicative effects represent concatenation of several singleton exposures, as direct effects, together with concurrency as an indirect effect. These concurrency and “hitchhiking” effects are otherwise unobservable within any one ICU patient and unidentifiable either within any one study or within any effect size whether derived from a single study or as a summary derived from several infection prevention intervention studies. These effects become apparent only by reference to an external benchmark.

Using the optimal model (Model A) for estimating the direct effects of various anti-fungal exposures reveals that topical amphotericin or anti-septic agents as singleton exposures would be estimated to each more than halve the incidence proportions of *Staphylococcus aureus* bacteremia and candidemia, although for absolute differences being approximately one percentage point or less. These small differences would be challenging to detect. For example, a cluster-randomized trial demonstrating halving in *Staphylococcus aureus* bacteremia incidence from 1% in the control group to 0.5% in the intervention group would need to enrol over 2,000 ICU’s each providing 500 patients per arm to provide 80% power [30].

The studies of SDD appear to show strong prevention effects as evident by a halving in *Staphylococcus aureus* VAP and candidemia and yet paradoxically, there is insignificant prevention of *Staphylococcus aureus* bacteremia (Table 1, Fig s2). Moreover, the incidences of candida and *Staphylococcus aureus* infections are generally higher among the concurrent control groups of antibiotic-based studies versus literature derived benchmarks (Figs. 2 & 3) and indicate strong contextual effects arising from concurrency which, in any one study, would be inapparent. In the optimal model (model A), these contextual effects are similar in size, but contrary in direction, to the modest direct effect of TAP on *Staphylococcus aureus* colonization [31–36].

Interaction between Candida and bacterial colonization underlying invasive infection demonstrated here resembles the findings from a similar causal model containing *Pseudomonas* colonization and infection as latent and measurement variables, respectively [14]. Also, the findings of the contextual effect of concurrency with TAP use within the ICU are similar to findings from a similar causal model containing *Pseudomonas* colonization and *Acinetobacter* colonization as latent variables [33]. The direct anti-bacterial effect of TAP is more evident within models containing either *Pseudomonas* or *Acinetobacter* colonization and infection data than is the case here with the model containing *Staphylococcus aureus* colonization and infection data. These four modelling studies are based on 328 studies, of which < 200 are common to all three [14, 33, 35].

The observations here could reconcile the contrary bacteremia prevention effects observed in large studies of combined antibiotic-and antifungal-based interventions using various SDD regimens. In studies where the SDD regimen containing topical polymyxin and tobramycin combined with amphotericin as the anti-fungal [10, 11], prevention effects were observed. By contrast, prevention effects were not observed in the largest studies to date, where the SDD intervention contained the same TAP regimen in combination with nystatin [12, 13]. The effect of concurrency could account for the striking difference in apparent prevention effects, being seemingly strong within studies of SDD and SOD using concurrent control groups, [18] which rely on an untested assumption that the concurrency effect is negligible, versus being either less evident or not observed within studies using non-concurrent control groups, [10, 12, 13, 37]. Rebound colonization on TAP withdrawal is also a difficult to quantify ecological effect [8, 38, 39].

Strengths of GSEM modelling are that it enables both generalized linear response functions and the ability to incorporate observations from clusters with missing observations under the assumption of missing at random. This enables the inclusion of groups from studies either lacking control groups or providing data for only some endpoints. Moreover, this analysis includes observations from a broad range of studies published over three and a half decades which have considerable heterogeneity in the interventions, exposures, populations, and study designs. These broadly selected studies provide the basis for a natural experiment with several simultaneous exposures [40].

## Limitations

The GSEM is a group level modelling of two latent variables, Candida colonization and *Staphylococcus aureus* colonization, within a postulated model of interaction leading to “hitchhiking.” These latent variables and the coefficients derived in the GSEM are indicative only. They have no counterpart at the level of any one patient or study. They indicate the propensity for invasive infection arising, by whatever mechanism, from colonization as a latent construct rather than colonization measured by its presence and density.

The second limitation is that the GSEM model is deliberately simplistic with most exposures coded as binary variables. The Candida species contributing to Candida colonization and candidemia have not been separately identified. There are limited numbers of key group level factors and the interaction between the latent variables being the only interactions tested. In reality, the relationships between expoures and outcomes will likely be graded and complex with potentially compound expoure interactions. The influence of topical placebo has not been considered here [36].

Thirdly, the various regimens of antibiotic-based, anti-septic, and anti-fungal interventions used within the various studies have been considered as similar within each category. This is a deliberate simplification. For example, some SAF interventions were administered parenterally rather than topically. Also, the intensity and duration of application, and the body site targeted by the various interventions, varied among the studies and have not been modelled. On the other hand, a strength of this analysis is that the various compound interventions, as for example within SDD regimens comprising TAP, PPAP, and anti-fungal components, are factorized towards estimating their separate singleton associations on the latent variables within the GSEM model.

Finally, with clustered data, the precision, as represented by the standard error estimates, is attenuated in comparison to what might have been possible if patient level data had been available.

## Conclusion

GSEM modelling of *Staphylococcus aureus* and candida colonization, each as latent variables versus antibiotic, anti-fungal, anti-septic, and various other group level exposures, demonstrates complex and paradoxical relationships that would not be apparent in any single study examined in isolation nor within the summary effect sizes of the respective interventions as derived by conventional meta-analytic modelling. The GSEM derived model (model A) validates the postulated interaction between candida and bacterial colonization in facilitating, by “hitchhiking,” invasive bacterial infections within clinical data derived from the ICU infection prevention literature. Anti-fungal interventions are projected to modestly prevent *Staphylococcus aureus* bacteremia, mediated via their effects on Candida in the colonizing flora.

## Supplementary Information

Below is the link to the electronic supplementary material.

Supplementary file1 (PDF 2453 KB)
