# Supplementary material for: Staphylococcus aureus hitchhiking from colonization to bacteremia via Candida within ICU infection prevention studies: a proof of concept modelling
Source: Eur J Clin Microbiol Infect Dis. 2023 Mar 6;42(5):543–54. doi: 10.1007/s10096-023-04573-1 (PMC10105687; doi:10.1007/s10096-023-04573-1)

Staphylococcus aureus hitchhiking from colonization to bacteremia via Candida within ICU infection prevention studies. A proof of concept modelling.

James C Hurley, MB BS, D Med Sci, M Epi, PhD, FRACP;

Author affiliations;

- Associate Professor, Melbourne Medical School, University of Melbourne,
- and Physician, Division of Internal Medicine, Ballarat Health Services, Ballarat, Victoria, Australia. e-mail jamesh@bhs.org.au

Supplemental file contents:

Table S1: Observational studies (Benchmark groups) 2 - 6

Table S2: Groups of non-decontamination studies 7- 10

Table S3: Groups of anti-septic studies 11 – 12

Table S4: Groups of antibiotic-based (= TAP ± PPAP ± antifungal) studies 13 – 18

Table S5: Groups from single drug anti-fungal (SAF) studies 19

Table S6: Review of effect sizes in the literature 20

References 21 – 39

Fig S1: Literature search and study group decant 40-41

Fig S2 Effect size *Staphylococcus* aureus bacteremia 42

Fig S3 Effect size Candidemia 43

Fig S4: GSEM model C 44

Fig S5: GSEM model B 45

Fig S6 a & b: Projections for *Staphylococcus aureus* bacteremia and Candidemia 46

Table S1: Observational studies (Benchmark groups) [a]

Author	Ref	Notes	MV	LOS	Patients	VAP		Blood stream infection	
			%	d	n	v_sr_n	v_can_n	b_sr_n	b_can_n
A'court 1993	1	T	100	12	150	4		5	
Alvarez-Lerma 1996	2		93	7	6494	102			
Antonelli 1994	3	T	70	17	124	10	1		
Apostolopoulou 2003	4		100	16	175	9			
Arumugam 2018	5	T	100	7	332	16			
Azoulay noCandida 2006	6		100	17	589				4
AzoulayCandida 2006	6	crf	100	17	214				2
Bailly 2015	7		100	30	1491				11
Bekaert 2011	8		100	8	4479	133			
Bercault 2001	9		100	26	1144		2		
Bercault_IHT 2005	10		100	9	118	2			
Bercault_noINT 2005	10	I	100	11	118	2			
Berrouane_all 1998	11	T	83	13	565	71			
Blot >74 2014	12		100	8	516	17			
Blot 45_64 2014	12		100	8	670	27			
Blot 65_74 2014	12		100	8	549	22			
Bochicchio 2004	13	T	100	13	678	50			
Bonten'94 1994	14		100	25	64	3			
Boots 2008	15		100	13	412	32	0		
Bornstain 2004	16		100	12	747	17			
Borzotta 1999	17	T	85	10	459				7
Braun 1986	18	T	100		66	6			
Bregeon 1997	19		100		660	34	3		
Bronchard 2004	20	T	100	23	109	26			
Cade 1993	21		98	16	98	13	5	4	1
Cavalcanti 2006	22	T	100	10	190	18	6		
Cenderero 1999	23		100	7	123	9	0		
Chaari 2015	24	T	100	8	175	6			
Charles 2005	25	crf	97	21	56				1
Charles 2005	25		75	14	36				0
Chastre 1998	26		100	14	243	18	7		
Chevret 1993	27		100	5	255	23	4		
Combes 2000	28	T	100	18	104	4			
Cook_non-trauma 2010	29		100	13	2080	14	4		
Cook_trauma 2010	29	T	100	8	511	15	1		
Craven-medical 1988	30		100	6	277	9	1	4	
Craven-surgical 1988	30		100	6	521	12	5	10	

Table S1 (continued): Observational studies (Benchmark groups)

Author	Ref	Notes	MV	LOS	Patients	VAP		Blood stream infection	
			%	d	n	v_sr_n	v_can_n	b_sr_n	b_can_n
Daschner 1988	31		100	6	116	13	4		
De waele 2003	32	crf	79	16	46				0
de_Latorre 1995	33		100	15	80	3	2		
de_Santis 2000	34				713			13	
de_Santis 2013	34				1318			0	
El-Masri 2004	35	T		11	361			24	
Ensminger 2006	36	C	100	7	92	6			
Ertugrul 2006	37	T	100	10	100	12		9	
Esteve 2007	38		80	16	395				
Esteve 2007	38		78	17	404				
Evans 2010	39	T	100	8	416	40			
Ewig 1999	40	T	100	10	48	5	1		
Fabian_all 1993	41	T	100	11	278	32			
Fagon'89 1989	42		100	13	567	17			
Ferreira 2015	43		94	5	2527				
Gacouin 2009	44		100	11	361	21			
Garcı´a-Garmendia 2001	45		46	5	2640			39	23
Garrouste-Orgeas 1997	46		100	11	86	13	1		
Garrouste-Orgeas 2006	47		75	11	3247			46	
George 1998	48		100	8	223	8	2		
Georges 2000	49		100	20	135	11			
Giamarellos-Bourboulis 2009	50	T	100	12	72		2	1	
Giard 2008	51		100	9	7236	193			
Gruson-97-98 2000	52	I	100		1029	54			
Gruson-95-96 2000	52		100		1004	67			
Gruson-99-01 2003	53		100		823	26			
Guérin 1997	54		100	19	260	3	0		
Gursel 2010	55		100	10	92	13	1		
Heyland 1999	56		100	7	1014	64	26		
Holzapfel_93 1993	57		100	10	300	8		6	
Huang_1pre 2013	58			3	15816			77	38
Huang_1SC 2013	58			3	23480			128	49
Hugonnet 2007	59		100	6	936	55	40		
Hyllienmark 2007	60		100	4	221	2			
Hyllienmark 2013	61	T	42	3	135	11			
Ibáñez 2000	62		100	8	30	3			
Ibrahim'00 2000	63		100	5	1882	143	19		
Ibrahim'00 2000	64		69	11	4913			94	41
Ibrahim'01 2001	65		56	9	880	36			
Jacobs 1990	66		100	15	24	2			
Jaillette 2011	67		100	15	439	22			

Table S1 (continued): Observational studies (Benchmark groups)

Author	Ref	Notes	MV	LOS	Patients	VAP		Blood stream infection	
			%	d	n	v_sr_n	v_can_n	b_sr_n	b_can_n
Jensen_HE 2015	68		66	6	604				24
Jensen_SOC 2015	68		67	5	596				13
Jimenez 1989	69		100	10	77	2			
Kallel 2020	70		93	26	2353			57	10
Kautzky 2014	71		37	17	35		1		0
Kautzky 2014	71	crf	57	24	30		1		2
Ko 2013	72		100	23	1453			16	
Kollef' 93 1993	73		100	7	277	9			
Kollef '95 1995	74		100		300	22			
Kollef '95 1995	75		100	16	314	17			
Kollef '97 1997	76		100	8	521	25			
Kollef '97_pre 1997	77	C	90	4	353	5	1	5	5
Kollef '97_post 1997	77	C I	90	4	327	4	0	1	3
Koss– N 2001	78		100	14	87	3	10		
Koss– P 2001	78	I	100	11	66	4	3		
Kunac 2014	79		100		716	62		6	
Laggner 1989	80	T	100	11	32	0	0	1	0
Lambert 2011	81			5	119699			462	
Laupland 2002	82			5	1017			18	3
Laupland 2004	83		84	5	4473			45	19
León 2006	84	crf	95	21	1699				58
León 2009	85	crf	91	17	1107				37
León 2016	86	crf	84	15	233				11
Lepelletier 2010	87	T	100	18	161	34			
Li 2016	88		17	12	29		1		0
Li 2016	88	crf	40	25	82		10		3
Luna 2003	89		100	8	427	19	2		
Luyt 2005	90		100		290	12			
Magnason 2008	91		100	8	280	1	0	1	5
Mahul 1992	92		100	22	145	10	3		
Makris 2011	93	I	100	22	152	3			
Markowicz 2000	94		100	10	744	74			
Memish 2000	95		100	11	202	16	4		
Michel 2005	96		100	11	299	12			
Mitsogianni 2011	97			16	124			0	1
Mitsogianni 2010	97			16	143			1	0

Table S1 (continued): Observational studies (Benchmark groups)

Author	Ref	Notes	MV	LOS	Patients	VAP		Blood stream infection	
			%	d	n	v_sr_n	v_can_n	b_sr_n	b_can_n
Moine 2002	98		80	14	764	19	2		
Montecalvo_J 1992	99		100	10	38			3	1
Myny 2005	100		100	4	385	27			
Nguile-Makao 2010	101		100	7	2873	89			
Nielsen 1992	102		100	5	242	5			
Nseir 2005	103		100	10	1241	15			
Nseir 2007	104	crf	100	24	102				3
Orsi 2007	105		98	36	1741			37	10
Orsi 2012	105		100	34	1165			4	4
Osmon 2003	106		72	8	893			29	26
Outcomerea 2019	107		100	8	7735	258			
Papazian 1996	108		100	10	586	20			
Petri 1997	109	crf	95	11	409				3
Potgieter 1987	110		78	9	250	23	5		
Prowle 2011	111		69	6	6339			88	51
Ramirez 2016	112		100	13	440	8	0		
Rello'91 1991	113		100	8	264	15	1		
Rello'92 1992	114	T	80	8	208	22			
Rello'92 1992	115		67	9	161	21			
Rello'94 1994	116		72		1650			16	4
Rello'02 2002	117		100	8	9080	143			
Rello'03 2003	118		100	20	99	2	0		
Resende 2013	119		100	22	126	5	0		
Reusser 1989	120		100	7	40	6		1	
Rincón-Ferrari 2004	121	T	100	10	310	27			
Rodrigues 2009	122		100	10	233	11	2		
Rodriguez 1991	123	T	100	14	294	37			
Ruiz-Santana 1987	124		100	7	1005	12	1		
Salata 1987	125		100	11	51	2	1		
Shahin 2013	126		100	10	267	6	3		
Sofianou 2000	127		100	36	198	13			
Stéphan 2006	128	T	100	16	175	43			
Tan 2016	129	crf	100	16	264	10			
Tan 2016	129		100	13	354	13			
Tejada-Artigas 2001	130	T	100	12	103	11			

Table S1 (continued): Observational studies (Benchmark groups)

Author	Ref	Notes	MV	LOS	Patients	VAP		Blood stream infection	
			%	d	n	v_sr_n	v_can_n	b_sr_n	b_can_n
Thompson 2008	131			6	4270			74	24
Timsit 1996	132		100	19	387	18			
Torres 1990	133		100	3	322	2			
Trouillet 1998	134		100		498	52			
Urli 2002	135		95	21	178	40	1	12	4
Valles 2007	136		100	22	60	9			
Vanhems 2011	137		100	6	3387	137			
Verhamme 2007	138		84	8	4000	56	6		
Violan 1998	139		100	16	314	26			
Warren 2001	140		28	4	3163			3	4
Woske 2001	141		100	19	103	29			
Xie 2011	142		100	7	4155	92	88		
Xie 2020	143		67	25	8474			45	45
Zahar 2009	144		100	10	1233	51			

Table S1 footnotes

Notes: T = Data originating from a study for which the majority of ICU admission were for trauma; C = cardio-thoracic ICU; I = Infection control intervention to entire ICU; crf = group wide candidemia risk factor

MV = percentage of group receiving mechanical ventilation; NS – Not stated; LOS is mean or median length of ICU stay; ICU-LOS is the ICU length of stay.

v_sr_n is the count of *Staphylococcus aureus* VAP; and v_can_n is the count of *Candida* isolates from patients with VAP.

b_sr_n is the count of *Staphylococcus aureus* bacteremia; b_can_n is the count of Candidemia;

Several (n = 43) of these studies were cited in the following source systematic reviews.

- Melsen WG, Rovers MM, Bonten MJM: Ventilator-associated pneumonia and mortality: A systematic review of observational studies. *Crit Care Med* 2009, 37:2709–2718.
- Safdar N, Dezfulian C, Collard HR, Saint S: Clinical and economic consequences of ventilator-associated pneumonia: a systematic review. *Crit Care Med* 2005, 33:2184–93.
- Agrafiotis M, Siempos II, Ntaidou TK, Falagas ME. Attributable mortality of ventilator-associated pneumonia: a meta-analysis. *Intern J Tub Lung Dis.* 2011;15(9):1154-1163.

Table S2: Groups of non decontamination studies [a]

Author	Ref	Notes	MV	LOS	Patients	VAP		Blood stream infection	
			%	d	n	v_sr_n	v_can_n	b_sr_n	b_can_n
Acosta-escribano 2010	145	T	100	18	54	4	1		
Acosta-escribano 2010	145	T	100	16	50	8	0		
Bonten 1995	146		100	17	67	4			0
Bonten 1995	146		100	19	74	7			0
Cook 1998	147		100	9	596	44	19		
Cook 1998	147		100	9	604	36	11		
Daumal 1999	148		100	6	174	7	2		
Daumal 1999	148		100	7	187	9	6		
Djedaini 1995	149		100	10	61	0	0		
Djedaini 1995	149		100	9	68	2	1		
Drakulovic 1999	150		100	10	47	4	0		
Drakulovic 1999	150		100	9	39	0	0		
Dreyfuss 1991	151		100	10	35	2			
Dreyfuss 1991	151		100	13	28	1			
Dreyfuss 1995	152		100	10	70	2			
Dreyfuss 1995	152		100	13	61	0			
Driks 1987	153		100	14	69	4	0		
Driks 1987	153		100	11	61	0	0		
Forestier 2008	154	T	100	13	106	11			
Forestier 2008	154	T	100	13	102	12			
Heyland 1999	155		100	12	46	0	0		
Heyland 1999	155		100	13	49	1	0		
Holzapfel_C 1999	156		100	15	200	21	3	2	1
Holzapfel_I 1999	156		100	17	199	7	1	2	1
Kappstein 1991	157	T	100	5	55	9	7		
Kappstein 1991	157	T	100	5	49	11	3		
Kirschenbaum 2002	158		100	20	20	1			
Kirschenbaum 2002	158		100	21	17	0			
Kirton 1997	159	T	100		140	6			
Kirton 1997	159	T	100		140	6			
Knight 2009	160		100	7	129	1	0		
Knight 2009	160		100	6	130	0	0		
Kollef 2008	161		100	4	743	16	7		
Kollef_silverETT 2008	161		100	4	766	9	5		
Lacherade 2005	162		100	25	184	16	2		
Lacherade 2005	162		100	21	185	18	0		

Table S2 (continued): Groups of non decontamination studies [a]

Author	Ref	Notes	MV	LOS	Patients	VAP		Blood stream infection	
			%	d	n	v_sr_n	v_can_n	b_sr_n	b_can_n
Lacherade 2010	163		100	11	164	8			
Lacherade 2010	163		100	11	169	2			
Laueny 2014	164	T	100	17	91	6	0		
Laueny 2014	164	T	100	11	98	17	0		
Lorente 2003	165		100	18	116	8	3		
Lorente 2003	165		100	16	114	7	4		
Lorente 2004	166		100	16	143	6	3		
Lorente 2004	166		100	20	161	14	2		
Lorente2006	167		100	13	233	11	1		
Lorente 2006	167		100	13	210	10	2		
Lorente'06a 2005	168		100	10	221	8	1		
Lorente'06a 2005	168		100	10	236	8	1		
Lorente 2006	169		100	21	51	5	0		
Lorente 2006	169		100	20	53	2	0		
Lorente 2007	170		100	16	140	8	0		
Lorente 2007	170		100	14	140	2	0		
Lorente 2014	171		100	16	150	5			
Lorente 2014	171		100	15	134	1			
Manzano 2008	172		100	12	63	9			
Manzano 2008	172		100	9	64	4			
Martin 1993	173		100	10	66	1	0		
Martin 1993	173		100	10	65	0	1		
Morrow 2010	174		100	15	73	14	1		
Morrow 2010	174		100	15	73	8	0		
Nseir 2011	175		100	10	61	3			
Nseir 2011	175		100	12	61	1			
Pickworth 1993	176	T	100	7	44	1	0		
Pneumatikos 2006	177	T	100	16	40	4	0		
Pneumatikos 2006	177	T	100	15	39	2	0		
Prod'hom_A 1994	178		100	6	81	5			
Prod'hom_S 1994	178		100	5	83	2			
Prod'hom_R 1994	178		100	5	80	4			
Reigneir 2013	179		100	10	222	17			
Reigneir 2013	179		100	10	227	10			
Rumbak 2004	180		100	5	60	5			
Rumbak 2004	180		100	16	60	1			

Table S2 (continued): Groups of non decontamination studies [a]

Author	Ref	Notes	MV	LOS	Patients	VAP		Blood stream infection	
			%	d	n	v_sr_n	v_can_n	b_sr_n	b_can_n
Ryan_C 1993	181		100	5	56	1			
Ryan_S 1993	181		100	6	58	2			
Smulders 2002	182		100	14	75	3	1		
Smulders 2002	182		100	12	75	1	0		
Staudinger 2010	183		100	14	75	2	2		
Staudinger 2010	183		100	8	75	2	0		
Thomachot 1998	184		100	12	66	7	0		
Thomachot 1998	184		100	12	70	8	0		
Thomachot 1999	185	T	100	11	77	8	0		
Thomachot 1999	185	T	100	12	63	7	0		
Thomachot 2002	186	T	100	9	84	7			
Thomachot 2002	186	T	100	9	71	5			
Valencia 2007	187		100	13	69	2			
Valencia 2007	187		100	13	73	2			
Walaszek 2017	188		100	5	804	4	5		
Walaszek 2017	188		100	5	1003	2	1		
Zeng 2016	189		100	22	117	16	4		
Zeng 2016	189		100	18	118	12	2		

Table S2 footnotes

Notes; T = Data originating from a study for which the majority of ICU admission were for trauma; C = cardio-thoracic ICU; crf = group wide candidemia risk factor;

MV = percentage of group receiving mechanical ventilation; NS – Not stated; LOS is mean or median length of ICU stay; ICU-LOS is the ICU length of stay.

v_sr_n is the count of *Staphylococcus aureus* VAP; and v_can_n is the count of *Candida* isolates from patients with VAP.

b_sr_n is the count of *Staphylococcus aureus* bacteremia; b_can_n is the count of Candidemia;

Several (n = 47) of these studies were cited in the following source systematic reviews.

- Messori A, Trippoli S, Vaiani M, Gorini M, Corrado A: Bleeding and pneumonia in intensive care patients given ranitidine and sucralfate for prevention of stress ulcer: meta-analysis of randomised controlled trials. *BMJ* 2000, 321:1103–1106.
- Huang J, Cao Y, Liao C, Wu L, Gao F: Effect of histamine-2-receptor antagonists versus sucralfate on stress ulcer prophylaxis in mechanically ventilated patients: a meta-analysis of 10 randomized controlled trials. *Crit Care* 2010, 14:R194.
- Alhazzani W, Almasoud A, Jaeschke R, Lo BW, Sindi A, Altayyar S, Fox-Robichaud A: Small bowel feeding and risk of pneumonia in adult critically ill patients: a systematic review and meta-analysis of randomized trials. *Crit Care* 2013, 17:R127.

- Melsen WG, Rovers MM, Bonten MJM: Ventilator-associated pneumonia and mortality: A systematic review of observational studies. *Crit Care Med* 2009, 37:2709–2718.
- Safdar N, Dezfulian C, Collard HR, Saint S: Clinical and economic consequences of ventilator-associated pneumonia: a systematic review. *Crit Care Med* 2005, 33:2184–93.
- Han J, Liu Y. Effect of ventilator circuit changes on ventilator-associated pneumonia: a systematic review and meta-analysis. *Respiratory care*, 2010; 55: 467-474.
- Subirana M, Solà I, Benito S: Closed tracheal suction systems versus open tracheal suction systems for mechanically ventilated adult patients. *Cochrane Database Syst Rev* 2007, 4: CD004581;
- Siempos II, Vardakas KZ, Kopterides P, Falagas ME. Impact of passive humidification on clinical outcomes of mechanically ventilated patients: A meta-analysis of randomized controlled trials. *Crit Care Med* 2007; 35: 2843-51;
- Muscedere J, Rewa O, McKechnie K, Jiang X, Laporta D, Heyland DK. Subglottic secretion drainage for the prevention of ventilator-associated pneumonia: a systematic review and meta-analysis. *Crit Care Med* 2011; 39:1985–1991.
- Delaney A, Gray H, Laupland KB, Zuege DJ. Kinetic bed therapy to prevent nosocomial pneumonia in mechanically ventilated patients: a systematic review and meta-analysis. *Crit Care* 2006; 10:R70;
- Sud S, Friedrich JO, Taccone P, Polli F, Adhikari NK, Latini R, Gattinoni L. Prone ventilation reduces mortality in patients with acute respiratory failure and severe hypoxemia: systematic review and meta-analysis. *Inten Care Med* 2010; 36(4); 585-599.
- Siempos II, Vardakas KZ, Falagas ME. Closed tracheal suction systems for prevention of ventilator-associated pneumonia. *Brit J Anaesthesia*, 2008; 100(3): 299-306.

Table S3: Groups of anti-septic studies [a]

Author & regimen	Ref	Notes	MV	LOS	Patients	VAP		Blood stream infection	
			%	d	n	v_sr_n	v_can_n	b_sr_n	b_can_n
Bellissimo-Rodrigues 2014	190		76	11	127		1		
Bellissimo-Rodrigues 2014_Chlx	190		77	11	127		0		
Bleasdale 2007	191		35	3	445			1	2
Bleasdale 2007_Chlx	191		36	3	391			0	2
Cabov 2010	192		57	6	30	2		2	
Cabov 2010_Chlx	192		77	6	30	0		0	
Caruso 2009	193		100	17	132	5	3		
Caruso 2009_Saline	193		100	17	130	1	0		
Climo_NC 2013	194			6	1398			8	16
Climo 2013_Chlx	194			6	1410			9	7
Fourrier 2000	195		100	24	30	3	1	1	0
Fourrier 2000_Chlx	195		100	18	30	0	0	0	0
Fourrier 2005	196		100	13	114	2	0	0	0
Fourrier 2005_Chlx	196		100	14	114	1	0	1	0
Huang_2pre 2013	58			3	15218			70	56
Huang_3pre 2013	58			3	17356			80	59
Huang_3UD 2013_Chlx	58			3	26024			92	62
Huang_2TD 2013_Chlx	58			3	24752			106	63
Koeman 2006	197		100	13	130	5	1		
Koeman 2006_Chlx	197		100	14	127	2	3		
Koeman 2006_ChlxC	197		100	13	128	5	4		
Kollef 2006	198		100	14	347	25	6		
Kollef 2006_Iseganin	198		100	14	362	17	0		
Lorente 2012	199		100	9	219	4	0		
Lorente 2012_Chlx	199		100	10	217	4	0		
Milstone 2013	200	P		3	1326			4	6
Milstone 2013_Chlx	200	P		3	667			3	3
Mori H 2006	201		100	7	414	5	2		
Mori 2006-PVI	201		100	8	1248	7	1		
Noto_pre 2015	202			3	4852			15	6
Noto_post 2015_Chlx	202			3	4488			16	2
Seguin – SC 2006	203	T	100	14	31	7			
Seguin – CC 2006	203	T	100	19	31	7			
Seguin 2006-PVI	203	T	100	15	36	3			

Table S3 (continued): Groups of anti-septic studies [a]

Author & regimen	Ref	Notes	MV	LOS	Patients	VAP		Blood stream infection	
			%	d	n	v_sr_n	v_can_n	b_sr_n	b_can_n
Seguin 2014	204	T	100	16	72	11			
Seguin 2014-PVI	204	T	100	15	78	14			
Swan 2016	205		57	7	164	2	1	0	
Swan 2016_Chlx	205		69	7	161	1	0	0	
Wittekamp 2018	206		100	10	2251			13	15
Wittekamp 2018_Chlx	206		100	10	2108			25	22

Table S3: Footnotes

Notes; T = Data originating from a study for which the majority of ICU admission were for trauma; P = paediatric ICU; I = Infection control intervention to entire ICU; crf = group wide candidemia risk factor;

MV = percentage of group receiving mechanical ventilation; NS – Not stated; LOS is mean or median length of ICU stay; ICU-LOS is the ICU length of stay.

v_sr_n is the count of *Staphylococcus aureus* VAP; and v_can_n is the count of *Candida* isolates from patients with VAP.

b_sr_n is the count of *Staphylococcus aureus* bacteremia; b_can_n is the count of Candidemia;

Several (n = 5) of these studies were cited in the following source meta-analyses.

- Chan EY, Ruest A, Meade MO, Cook DJ: Oral decontamination for prevention of pneumonia in mechanically ventilated adults: systematic review and meta-analysis. *BMJ* 2007, 334:889–900.
- Labeau SO, Van de Vyver K, Brusselaers N, Vogelaers D, Blot SI: Prevention of ventilator-associated pneumonia with oral antiseptics: a systematic review and meta-analysis. *Lancet Infect Dis* 2011, 11:845-854.
- Pileggi C, Bianco A, Flotta D, *et al*. Prevention of ventilator-associated pneumonia, mortality and all intensive care unit acquired infections by topically applied antimicrobial or antiseptic agents: a meta-analysis of randomized controlled trials in intensive care units. *Crit Care.* 2011; **15**: R155.
- Price R, MacLennan G, Glen J. Selective digestive or oropharyngeal decontamination and topical oropharyngeal chlorhexidine for prevention of death in general intensive care: systematic review and network meta-analysis. *BMJ*. 2014; **348**: g2197.
- Klompas M, Speck K, Howell MD, *et al*. Reappraisal of routine oral care with chlorhexidine gluconate for patients receiving mechanical ventilation: systematic review and meta-analysis. *JAMA Intern Med.* 2014; **174**: 751-61.

Intervention regimens abbreviations

Chlx = chlorhexidine; Chlx BW = chlorhexidine body wash; ChC = chlorhexidine and colisitn; TD = targetted decolonization; UD = universal decolonization; PVI = povidone iodine; CC = concurrent control; SC = saline control; iseganan, is a synthetic variant of a porcine protegrin, which is a natural antibiotic peptide released by neutrophils in response to invasion by microbes [Kollef 2006].

Table S4: Groups of antibiotic-based prophylaxis (=TAP±PPAP±antifungal) studies [a]

Author & regimen	Ref	Notes	MV	LOS	Patients	VAP		Blood stream infection	
			%	d	n	v_sr_n	v_can_n	b_sr_n	b_can_n
Groups from NCC studies									
Bergmans NC 2001	207		100	12	61	5	1		
Bergmans CC 2001	207		100	15	78	6	3		
Bergmans 2001_PGV	207		100	13	87	3	1		
Bonten NC 1994	208		91	16	54	2			
Bonten CC 1994	208		86	9	21	0			
Bonten 1994_PTA	208		100	13	22	0			
Camus NC 2014	209		28	4	925	17	3	3	2
Camus 2014_PT	209		28	4	1022	0	1	2	1
De La Court 2021_PTA	210				722			7	4
De La Court 2021_PTA	210				1236			12	11
de Smet 2009	211		88	9	1990			22	16
de Smet SDD 2009_PTA	211		93	9	2045			9	8
de Smet SOD 2009_PTA	211		94	9	1904			9	14
Godard 1990	212		80	13	84	4			2
Godard 1990_PT	212		81	11	97	0			0
Gorensek 1993	213	crf		15	34			0	1
Gorensek 1993_NoNy	213	crf		15	17			0	0
Hartenauer 1990	214		100	14	101	8	0	3	0
Hartenauer 1990_PTA	214		100	13	99	3	1	4	3
Hjortrup 1997_CefTNy	215	crf	100		150	14	11	2	4
Konrad 1989	216		100		83	4			
Konrad 1989_PTA	216		100		82	2			
Landelle 2018	217		100	9	291	10			
Landelle 2018_PTNy	217		100	8	413	9			
Landelle 2018_PTNy	217		100	9	356	4			
Ledingham 1988	218		60	5	161	5			
Ledingham 1988_PTA	218		60	5	163	1			
Leone 2002_PTA	219	T	100	12	324	23			
Mathieu 2day 2020_PTA	220	T	100		199				0
Mathieu 3day 2020_PTA	220	T	100		248				1
Nardi 1990	221		100	13	50	3			
Nardi 1990_PTA	221		100	12	47	3			

Table S4 (continued): Groups of antibiotic-based prophylaxis (=TAP±PPAP±antifungal) studies [a]

Author & regimen	Ref	Notes	MV	LOS	Patients	VAP		Blood stream infection	
			%	d	n	v_sr_n	v_can_n	b_sr_n	b_can_n
Groups from NCC studies									
Nardi 2001_PTAM	222	T	100	11	119	1	0	2	
Nardi 2001_PTA	222	T	100	12	104	9	1	2	
Ong 2015_PTA	223		87	10	3080			10	
Oostdijk SDD 2014_PTA	224		51	6	5483			13	
Oostdijk SOD 2014_PTA	224		52	6	5508			32	
Rouby 1994_P	225		100	12	347	21			
Rouby 1994_E	225		100	18	251	14			
Silvestri 1999_PTA	226	e	100	9	117	2	0	8	
Steffen 1994_PTNy	227	crf	100	14	127			1	
Stoutenbeek 1984	228	T	100	14	59	18	0	0	
Stoutenbeek 1984_PTA	228	T	100	11	63	0	0	0	
Stoutenbeek 1987	229	T	100	14	59	3	0	7	
Stoutenbeek SDD 1987_PTA	229	T	100	11	63	3	0	0	
Stoutenbeek ED 1987_PTA	229	T	100	18	42	9	0	3	
SuDDICU 2022	230		100	11	3191			46	
SuDDICU 2022	230		100	10	2791			27	
Valles 2013	231		100	15	58	1			
Valles 2013	231		100	10	71	2			
Veelo 2008_PTA	232		100	17	231	8			
Winter CC 1992	233		92	8	92	1	0		
Winter NC 1992	233		92	7	84	4	0		
Winter 1992_PTA	233		92	6	91	0	0		
Wittekamp 2018_ PTNy	206		100	10	2224			12	23
Wittekamp 2018_PTNy	206		100	11	2082			17	18

Table S4 (continued): Groups of antibiotic-based prophylaxis (=TAP±PPAP±antifungal) studies [a]

Author & regimen	Ref	Notes	MV	LOS	Patients	VAP		Blood stream infection	
			%	d	n	v_sr_n	v_can_n	b_sr_n	b_can_n
Groups from CC studies									
Abele-Horn 1997	234	T	100	22	30	5			
Abele-Horn 1997_ PTA	234	T	100	18	58	9			
Acquarolo 2005	235		100	13	19	6	0		
Acquarolo 2005_AmpSul	235		100	13	19	3	0		
Aerdts 1991	236		100	28	39	4	2		1
Aerdts 1991_ PNoA	236		100	23	17	0	0		0
Bion 1991	237	crf	50	2	31		2	0	0
Bion 1991_ PTA	237	crf	50	2	21		3	0	0
Blair 1991	238		93	8	130	7	1		0
Blair 1991_ PTA	238		93	8	126	1	0		1
Blaise 1994	239	crf	33	7	45	4		3	
Blaise 1994_O	239	crf	37	7	46	1		4	
Bouza 2013	240	C	100	12	38	4	0		
Bouza 2013_Lnz_Mrp	240	C	100	10	40	2	0		
Camus 2005	241		100	13	126	8		1	
Camus 2005_MChlx	241		100	10	130	3		1	
Camus 2005_ PT	241		100	12	130	6		3	
Camus 2005_ PTMCh	241		100	11	129	1		2	
Cerra 1992	242	e		26	21	8	10		4
Cerra 1992_NoNy	242	e		18	25	5	6		1
Cockerill 1992	243		85	12	75				2
Cockerill 1992_ PGNy	243		85	10	75				1
de la Cal 2005	244	T	80	34	54	10	0	6	0
de la Cal 2005_ PTA	244	T	74	31	53	0	2	14	1
Ferrer 1994	245		100	14	41	2	2	2	0
Ferrer 1994_ PTA	245		100	15	39	3	0	0	0
Flaherty 1990	246	C	40	4	56				0
Flaherty 1990_ PGA	246	C	40	4	51	0			0
Francois	247		100	11	95	8	0		
Francois	247		100	11	99	3	3		
Garbino_EN 2004_ PNeV	248		100	14	71		1		3
Garbino_TPN 2004_ PNeV	248		100	14	29		0		2

Table S4 (continued): Groups of antibiotic-based prophylaxis (=TAP±PPAP±antifungal) studies [a]

Author & regimen	Ref	Notes	MV	LOS	Patients	VAP		Blood stream infection	
			%	d	n	v_sr_n	v_can_n	b_sr_n	b_can_n
Gaussorgues 1991	249		100	17	59		1	2	15
Gaussorgues 1991_ PGA	249		100	16	59		0	1	5
Georges 1994	250	T	100	7	33	1	0		0
Georges 1994_ PNeA	250	T	100	7	31	0	0		0
Hammond 1994	251	T	100	14	33	3			
Hammond 1994_ PTA	251	T	100	16	39	4			
Jacobs 1992	252		100	10	43	0	0	0	0
Jacobs 1992_ PTA	252		100	9	36	0	0	0	0
Karvouniaris 2015	253		100	13	84	4			
Karvouniaris 2015_P	253		100	16	84	5			
Kerver 1988	254		100	20	47		0		0
Kerver 1988_ PTA	254		100	17	49		0		0
Korinek 1993	255	T	100	27	60	16	0		
Korinek 1993_ PTA	255	T	100	25	63	9	0		
Laggner 1994	256		100	20	34	0	0	0	0
Laggner 1994_ PA	256		100	16	33	0	0	1	0
Langlois-Karaga 1995	257	T	100	11	50	15			
Langlois-Karaga 1995_ PGA	257	T	100	11	47	5			
Mirateli 2019	258		100	46	42	3			
Mirateli 2019	258		100	33	42	2			
Palomar 1997	259		100	6	42	8	0		0
Palomar Ctx 1997	259		100	8	46	3			1
Palomar_1 1997	259		100	11	41	5	0		0
Quinio 1995	260	T	100	16	72	16	0	4	0
Quinio 1995_ PGA	260	T	100	16	76	9	0	8	0
Rimola 1985	261	crf		10	72			5	
Rimola 1985_ PGVNy	261	crf		11	68			2	
Rocha 1992	262	T	100	18	54	15		3	0
Rocha 1992_ PTA	262	T	100	19	47	5		2	0
Rodríguez-Roldán 1990	263		100	12	15	1			
Rodríguez-Roldán 1990_ PTNeA	263		100	10	13	0			

Table S4 (continued): Groups of antibiotic-based prophylaxis (=TAP±PPAP±antifungal) studies [a]

Author & regimen	Ref	Notes	MV	LOS	Patients	VAP		Blood stream infection	
			%	d	n	v_sr_n	v_can_n	b_sr_n	b_can_n
Rolando 1996	264	crf	73		61	1	1	1	1
Rolando 1996_ PTA	264	crf	73		47	2	1	0	0
Rolando 1993	265	crf	75	8	31	1	3	3	0
Rolando 1993_ PTAM	265	crf	75	8	28	2	0	0	0
Rolando_infected 1993	265	crf	75	8	21	0	1	0	0
Rolando_infected 1993_ PTAM	265	crf	75	7	21	1	1	0	0
Sanchez-Garcia 1998	266		100	20	140	7	0		
Sanchez-Garcia 1998_ PTA	266		100	17	131	5	0		
Sirvent 1997	267	T	100	16	50	11	0		
Sirvent 1997	267	T	100	13	50	3	0		
Smith 1993	268	crf	100	8	18		1		1
Smith 1993_ PTA	268	crf	100	7	18		0		0
Stoutenbeek 2007	269	T	100	12	200	40	21	5	0
Stoutenbeek 2007_ PTA	269	T	100	13	201	18	6	8	1
Ulrich 1989	270		83	13	52	5	1		0
Ulrich 1989_PNoA	270		77	17	48	2	1		0
Unertl 1987	271		100	23	20	5	0		
Unertl 1987_PGA	271		100	18	19	1	0		
van Delden 2012	272		100	11	45				
van Delden 2012_Azith	272		100	10	47				
Verwaest 1997	273		100	19	185	9	0	3	4
Verwaest 1997_ PTA	273		100	22	200	9	0	10	0
Verwaest 1997_OA	273		100	17	193	6	1	7	0
Wiener 1995	274		100	11	31	4	0		2
Wiener 1995_ PGNy	274		100	11	30	1	0		0
Zobel 1991	275	P	100	13	25			0	1
Zobel 1991_PGA	275	P	100	14	25			0	0

Table S4: Footnotes

Notes; T = Data originating from a study for which the majority of ICU admission were for trauma; C = cardio-thoracic ICU; P = paediatric ICU; crf = group wide candidemia risk factor; e = number of episodes rather than patients

MV = percentage of group receiving mechanical ventilation; NS – Not stated; LOS is mean or median length of ICU stay; ICU-LOS is the ICU length of stay.

v_sr_n is the count of *Staphylococcus aureus* VAP; and v_can_n is the count of *Candida* isolates from patients with VAP.

b_sr_n is the count of *Staphylococcus aureus* bacteremia; b_can_n is the count of Candidemia;

The control group in one study by Stoutenbeek [1987] appears also as the control group in another study by this Author [1984] and is used only once in the analysis here.

Several (n = 24) of these studies were cited in the following source systematic reviews.

- Minozzi S, Pifferi S, Brazzi L, Pecoraro V, Montrucchio G, D'Amico R. Topical antibiotic prophylaxis to reduce respiratory tract infections and mortality in adults receiving mechanical ventilation. Cochrane Database Syst Rev 2021, Issue 1. Art. No.: CD000022. DOI: 10.1002/14651858.CD000022.pub4.
- Pileggi C, Bianco A, Flotta D, Nobile CG, Pavia M. Prevention of ventilator-associated pneumonia, mortality and all intensive care unit acquired infections by topically applied antimicrobial or antiseptic agents: a meta-analysis of randomized controlled trials in intensive care units. *Crit Care* 2011; 15:R155.
- Silvestri L, Van Saene HK, Milanese M, Gregori D, Gullo A. Selective decontamination of the digestive tract reduces bacterial bloodstream infection and mortality in critically ill patients. Systematic review of randomized, controlled trials. J Hosp Infect. 2007;65(3):187-203.

TAP intervention regimens abbrevations; PTA (=P, topical polymyxin; T, topical tobramycin; A, topical amphotericin); PTA-Ctx (=P, topical polymyxin; T, topical tobramycin; A, topical amphotericin; Ctx, parenteral cephalosporin); P (P = polymyxin either aerosolized or topical); PNeV (P = polymyxin; Ne = Neomycin; V = Vancomycin); PGA-Ctx (=P, topical polymyxin; G, topical gentamicin; A, topical amphotericin; Ctx, parenteral cephalosporin); PTAM (=P, topical polymyxin; T, topical tobramycin; A, topical amphotericin; topical mupirocin); E (=E, topical erythromycin); PNoA-Ctx (=P, topical polymyxin; No, topical norfloxacin; A, topical amphotericin; Ctx, parenteral cephalsporin); PGV (=P, topical polymyxin; G, topical gentamicin; V, topical vancomycin); PGNy-Ctx (=P, topical polymyxin; G, topical gentamicin; Ny, topical nystatin; Ctx, parenteral cephalsporin); P-Ctx (=P, topical polymyxin; Ctx, parenteral cephalosporin); PTAV (=P, topical polymyxin; T, topical tobramycin; A, topical amphotericin; V, topical vancomycin); PGA (=P, topical polymyxin; G, topical gentamicin; A, topical amphotericin); PTNeA (=P, topical polymyxin; T, topical tobramycin; Ne, topical Neomycin; A, topical amphotericin); PGNy (=P, topical polymyxin; G, topical gentamicin; Ny, topical nystatin); PTA-Cz (=P, topical polymyxin; T, topical tobramycin; A, topical amphotericin; Cz, parenteral Ceftazidime).

Table S5: Groups of studies of single drug antifungal (SAF) prophylaxis

Author & regimen	Ref	Notes	MV	LOS	Patients	RT Candida	Candidiemia
			%	d	n	v_can_n	b_can_n
Ables 2000	276	crf	95	8	59		0
Ables 2000_Fluc	276	crf	95	8	60		0
Eggimann 1999	277	crf			20		2
Eggimann 1999_Fluc	277	crf			23		0
Garbino 2002	278		100	8	101	0	9
Garbino 2002_Fluc	278		100	9	103	2	1
Giglio 2012	279		100	15	50	0	0
Giglio 2012_Ny	279		100	15	49	0	0
Jacobs 2003	280			11	39		1
Jacobs 2003_Fluc	280			11	32		0
Lumbreras 1996_Ny	281	crf		28	67		0
Lumbreras 1996_Fluc	281	crf		28	76		1
Normand 2005	282		100	12	47	0	0
Normand 2005_Ny	282		100	12	51	0	0
Ostrosky-Zeichner 2014	283	crf	100	7	102		7
Ostrosky-Zeichner 2014_Casp	283	crf	100	7	117		1
Parizkova 2000	284		100		20		0
Parizkova 2000_Fluc	284		100		18		0
Pelz_2001	285	T	87	5	130		3
Pelz_2001_Fluc	285	T	88	5	130		1
Piarroux_2004	286			15	455		6
Piarroux_2004_Fluc	286			12	428		0
Savino 1994	287		31	16	72		0
Savino 1994_Clot	287		23	12	80		0
Savino 1994_Ket	287		33	8	65		0
Savino 1994_Ny	287		39	15	75		0
Savino 1994	287	crf	31	16	72		2
Savino 1994_Clot	287	crf	23	12	80		1
Savino 1994_Ket	287	crf	33	8	65		1
Savino 1994_Ny	287	crf	39	15	75		5
Schuster 2008	288	crf		12	127		2
Schuster 2008_Fluc	288	crf		12	122		0

Table S5: Footnotes

T = Data originating from a trauma ICU; crf = group wide candidemia risk factor; MV = percentage of group receiving mechanical ventilation; NS – Not stated; LOS is mean or median length of ICU stay; ICU-LOS is the ICU length of stay.

RT Candida is respiratory tract candida (v_can_n; the count of *Candida* isolates from patients with VAP); b_can_n is the count of Candidemia

Several (n = 24) of these studies were cited in the following source systematic reviews [286-296].

Table S6: Review of effect sizes in the literature [a, b]

	Effect size	95% CI	notes	Ref
**Anti-septics**				
Overall VAP				
	RR: 0.78	0.60 – 1.02 (13)	Excl cardiac surgery	290
	RR: 0.67	0.47 – 0.97 (13)	Chlorhexidine	291
	RR: 0.66	0.51 – 0.85 (22)	Nosocomial pneumonia	292
Overall bactremia				
	OR: 0.74	0.37 – 1.50 (5)	Chlorhexidine	293
**Antibiotic based**				
Overall VAP				
	OR: 0.35	0.30 – 0.42 (26)		294
	RR: 0.43	0.35 – 0.53 (18)	TAP + PPAP	295
	RR: 0.57	0.44 – 0.74 (19)	TAP alone (incl duplex)	295
	RR: 0.59	0.36 – 0.69 (13)	TAP alone (excl duplex)	295
Bacteraemia				
	OR: 0.48	0.37 – 0.64 (16)	Overall bacteraemia	294
	OR: 0.30	0.16 – 0.56 (8)	Gram Neg (TAP + PPAP)	296
	OR: 1.04	0.69 – 1.57 (8)	Gram Pos (TAP + PPAP)	296
	OR: 0.63	0.46 – 0.87 (16)	Overall bacteremia	296
Candida infections				
	OR: 0.39	0.21 – 0.72 (11)	Candidemia	297
	OR: 0.29	0.18 – 0.45 (15)	Invasive fungal infections	297
	OR: 0.32	0.18 – 0.57 (5)	(Overall fungal infections)	298
**Single anti-fungal (SAF)**				
Candidemia				
	OR: 0.32	0.12 – 0.82 (6)		297
Other candida infections				
	OR: 0.54	0.39 – 0.75 (10)	Invasive fungal infections	297
	OR: 0.69	0.37 – 1.79 (5)	superficial fungal infections	299
	OR: 0.57	0.39 – 0.83 (18)	Invasive fungal infections	299
	OR: 0.71	0.52 – 0.97 (12)	fungal colonization	299
	RR: 0.46	0.31 – 0.68 (9)	Invasive fungal infections	300

Table S6: Footnotes

OR = Odds ratio; RR = Risk ratio

References

S1. A'Court CH, Garrard CS, Crook D, Bowler I, Conlon C, Peto T, Anderson E: Microbiological lung surveillance in mechanically ventilated patients, using non-directed bronchial lavage and quantitative culture. Q J Med. 1993;86:635-48.

S2. Alvarez-Lerma F, ICU-acquired Pneumonia Study Group. Modification of empiric antibiotic treatment in patients with pneumonia acquired in the intensive care unit. *Intens Care Med.* 1996;22(5):387-94.

S3. Antonelli M, Moro ML, Capelli O, De Blasi RA, D'Errico RR, Conti G, Bufi M, Gasparetto A: Risk factors for early onset pneumonia in trauma patients. Chest. 1994;105:224-228

S4. Apostolopoulou E, Bakakos P, Katostaras T, Gregorakos L: Incidence and risk factors for ventilator-associated pneumonia in 4 multidisciplinary intensive care units in Athens, Greece. Respir Care. 2003;48: 681-688.

S5. Arumugam SK, Mudali I, Strandvik G, El-Menyar A, Al-Hassani A, Al-Thani H. Risk factors for ventilator-associated pneumonia in trauma patients: A descriptive analysis. World J Emerg Med. 2018;9(3):203.

S6. Azoulay E, Timsit JF, Tafflet M, de Lassence A, Darmon M, Zahar JR, Schlemmer B. *Candida* colonization of the respiratory tract and subsequent pseudomonas ventilator-associated pneumonia. *Chest* 2006;129(1):110-117.

S7. Bailly S, Bouadma L, Azoulay E, Orgeas MG, Adrie C, Souweine B, Schwebel C, Maubon D, Hamidfar-Roy R, Darmon M, Wolff M. Failure of empirical systemic antifungal therapy in mechanically ventilated critically ill patients. *Am J Respir Crit Care Med.* 2015;191(10):1139-46.

S8. Bekaert M, Timsit JF, Vansteelandt S, Depuydt P, Vésin A, Garrouste-Orgeas M, Decruyenaere J, Clec'h C, Azoulay E, Benoit D. Attributable mortality of ventilator-associated pneumonia: a reappraisal using causal analysis. *Am J Respir Crit Care Med*. 2011;184(10):1133-9.

S9. Bercault N, Boulain T: Mortality rate attributable to ventilator-associated nosocomial pneumonia in an adult intensive care unit: a prospective case-control study. Crit Care Med. 2001;29:2303-2309

S10. Bercault N, Wolf M, Runge I, et al. Intrahospital transport of critically ill ventilated patients: a risk factor for ventilator-associated pneumonia-a matched cohort study. *Crit Care Med* 2005;33:2471-8.

S11. Berrouane Y, Daudenthun I, Riegel B, Emery MN, Martin G, Krivosic R, Grandbastien B. Early onset pneumonia in neurosurgical intensive care unit patients. J Hosp Infect. 1998;40(4):275-80.

S12. Blot S, Koulenti D, Dimopoulos G, Martin C, Komnos A, Krueger WA, Spina G, Armaganidis A, Rello J. Prevalence, risk factors, and mortality for ventilator-associated pneumonia in middle-aged, old, and very old critically ill patients. Crit Care Med. 2014;42(3):601-9.

S13. Bochicchio GV, Joshi M, Bochicchio K, Tracy K, Scalea TM: A time-dependent analysis of intensive care unit pneumonia in trauma patients. J Trauma. 2004;56:296-301.

S14. Bonten MJ, Gaillard CA, van Tiel FH, Smeets HG, van der Geest S, Stobberingh EE: The stomach is not a source for colonization of the upper respiratory tract and pneumonia in ICU patients. Chest. 1994;105(3):878-84.

S15. Boots RJ, Phillips GE, George N, Faoagali JL. Surveillance culture utility and safety using low-volume blind bronchoalveolar lavage in the diagnosis of ventilator-associated pneumonia. Respirology. 2008;13:87-96.

S16. Bornstain C, Azoulay E, De Lassence A, Cohen Y, Costa MA, Mourvillier B, Descorps-Declere A, Garrouste-Orgeas M, Thuong M, Schlemmer B, Timsit JF: Sedation, sucralfate, and antibiotic use are potential means for protection against early-onset ventilator-associated pneumonia. Clin Infect Dis. 2004;38(10):1401-8.

S17. Borzotta AP, Beardsley K (1999) Candida infections in critically ill trauma patients: a retrospective case-control study. Arch Surg 134(6):657-665

S18. Braun SR, Levin AB, Clark KL. Role of corticosteroids in the development of pneumonia in mechanically ventilated head-trauma victims. Crit Care Med 1986;14:198-201

S19. Bregeon F, Papazian L, Visconti A, Gregoire R, Thirion X, Gouin F: Relationship of microbiologic diagnostic criteria to morbidity and mortality in patients with ventilator-associated pneumonia. JAMA. 1997;277: 655-662

S20. Bronchard R, Albaladejo P, Brezac G, et al. Early onset pneumonia: risk factors and consequences in head trauma patients. *Anesthesiology* 2004;100:234-9.

S21. Cade JF, McOwat E, Siganporia R, Keighley C, Presneill J, Sinickas V: Uncertain relevance of gastric colonization in the seriously ill. Intensive Care Med. 1992;18:210-217

S22. Cavalcanti M, Ferrer M, Ferrer R, Morforte R, Garnacho A, Torres A: Risk and prognostic factors of ventilator-associated pneumonia in trauma patients. Crit Care Med. 2006;34:1067-1072

S23. Cendrero JA, Solé-Violán J, Benitez AB, Catalán JN, Fernández JA, Santana PS, de Castro FR: Role of different routes of tracheal colonization in the development of pneumonia in patients receiving mechanical ventilation. Chest. 1999;116:462-470

S24. Chaari A, El Habib M, Ghdhoun H, Algia NB, Chtara K, Hamida CB, Chelly H, Bahloul M, Bouaziz M. Does low-dose hydrocortisone therapy prevent ventilator-associated pneumonia in trauma patients? Am J Therap. 2015;22(1):22-8.

S25. Charles PE, Dalle F, Aube H, Doise JM, Quenot JP, Candida spp. colonization significance in critically ill medical patients: a prospective study. Intensive Care Med 2005; 31(3):393-400.

S26. Chastre J, Trouillet JL, Vuagnat A, Joly-Guillou ML, Clavier H, Dombret MC, Gibert C: Nosocomial pneumonia in patients with acute respiratory distress syndrome. *Am J Respir Crit Care Med.* 1998;157:1165-1172

S27. Chevret S, Hemmer M, Carlet J: Incidence and risk factors of pneumonia acquired in intensive care units. Results from a multicenter prospective study on 996 patients. European Cooperative Group on Nosocomial Pneumonia. Intensive Care Med. 1993;19:256-264

S28. Combes P, Fauvage B, Oleyer C. Nosocomial pneumonia in mechanically ventilated patients, a prospective randomised evaluation of the Stericath closed suctioning system. *Intensive Care Med* 2000;26:878-82.

S29. Cook A, Norwood S, Berne J: Ventilator-associated pneumonia is more common and of less consequence in trauma patients compared with other critically ill patients. J Trauma Acute Care Surg. 2010;69(5):1083-91.

S30. Craven DE, Kunches LM, Lichtenberg DA, Kollisch NR, Barry MA, Heeren TC, McCabe WR: Nosocomial infection and fatality in medical and surgical intensive care unit patients. Arch Intern Med. 1988;148:1161-1168

S31. Daschner F, Kappstein I, Schuster F, Scholz R, Bauer E, Jooβens D, Just H: Influence of disposable ('Conchapak') and reusable humidifying systems on the incidence of ventilation pneumonia. J Hosp Infect. 1988;11:161-168

S32. De Waele JJ, Vogelaers D, Blot S Colardyn F (2003) Fungal infections in patients with severe acute pancreatitis and the use of prophylactic therapy. Crit Care 2003: 7(2) 1.

S33. De Latorre FJ, Pont T, Ferrer A, Rosselló J, Palomar M, Planas M: Pattern of tracheal colonization during mechanical ventilation. Am J Respir Crit Care Med. 1995;152:1028-1033

S34. De Santis V, Gresoiu M, Corona A, Wilson AP, Singer M. Bacteraemia incidence, causative organisms and resistance patterns, antibiotic strategies and outcomes in a single university hospital ICU: continuing improvement between 2000 and 2013. J Antimicrob Chemoth. 2015;70(1):273-8.

S35. El-Masri MM, Hammad TA, McLeskey SW, Joshi M, Korniewicz DM. Predictors of nosocomial bloodstream infections among critically ill adult trauma patients. *Infect Cont & Hosp Epidemiol.* 2004;25(8):656-63.

S36. Ensminger SA, Wright RS, Baddour LM, Afess B: Suspected ventilator-associated pneumonia in cardiac patients admitted to the coronary care unit. Mayo Clin Proc. 2006;81:32–35

S37. Ertugrul BM, Yildirim A, Ay P, Oncu S, Cagatay A, Cakar N, Ertekin C, Ozsut H, Eraksoy H, Calangu S. Ventilator-associated pneumonia in surgical emergency intensive care unit. Saudi Med J. 2006;27(1):52.

S38. Esteve F, Pujol M, Limon E, Saballs M, Argerich MJ, Verdaguer R, Manez R, Ariza X, Gudiol F. Bloodstream infection related to catheter connections: a prospective trial of two connection systems. *J Hosp Infect*. 2007;67(1):30-4.

S39. Evans HL, Zonies DH, Warner KJ, Bulger EM, Sharar SR, Maier RV, Cuschieri J. Timing of intubation and ventilator-associated pneumonia following injury. Arch Surg. 2010;145(11):1041-6.

S40. Ewig S, Torres A, El-Ebiary M, Fàbregas N, Hernandez C, Gonzalez J, Nicolas JM, Soto L: Bacterial colonization patterns in mechanically ventilated patients with traumatic and medical head injury. Incidence, risk factors, and association with ventilator-associated pneumonia. Am J Respir Crit Care Med. 1999;159:188-198

S41. Fabian TC, Boucher BA, Croce MA, Kuhl DA, Janning SW, Coffey BC, Kudsk KA: Pneumonia and stress ulceration in severely injured patients: a prospective evaluation of the effects of stress ulcer prophylaxis. Arch Surg. 1993;128(2):185-92.

S42. Fagon JY, Chastre J, Domart Y, Trouillet JL, Pierre J, Darne C, Gibert C: Nosocomial pneumonia in patients receiving continuous mechanical ventilation. Prospective analysis of 52 episodes with use of a protected specimen brush and quantitative culture techniques. *Am Rev Respir Dis* 1989;139:877-884.

S43. Ferreira D, Grenouillet F, Blasco G, Samain E, Hénon T, Dussaucy A, Millon L, Mercier M, Pili-Floury S. Outcomes associated with routine systemic antifungal therapy in critically ill patients with Candida colonization. Intens care Med. 2015;41(6):1077-88.

S44. Gacouin A, Barbarot N, Camus C, Salomon S, Isslame S, Marque S, Lavoué S, Donnio PY, Thomas R, Le Tulzo Y. Late-onset ventilator-associated pneumonia in nontrauma intensive care unit patients. Anesth Analg. 2009;109(5):1584-90.

S45. García-Garmendia JL, Ortiz-Leyba C, Garnacho-Montero J, Jiménez-Jiménez FJ, Pérez-Paredes C, Barrero-Almodóvar AE, Miner MG. Risk factors for Acinetobacter baumannii nosocomial bacteremia in critically ill patients: a cohort study. *Clin Infect Dis*. 2001;33(7):939-46.

S46. Garrouste-Orgeas M, Chevret S, Arlet G, Marie O, Rouveau M, Popoff N, Schlemmer B: Oropharyngeal or gastric colonization and nosocomial pneumonia in adult intensive care unit patients. A prospective study based on genomic DNA analysis. Am J Respir Crit Care Med. 1997;156:1647-56.

S47. Garrouste-Orgeas M, Timsit JF, Tafflet M, Misset B, Zahar JR, Soufir L, Carlet J: Excess risk of death from intensive care unit—acquired nosocomial bloodstream infections: a reappraisal. *Clin Infect Dis* 2006, 42:1118-1126.

S48. George DL, Falk PS, Wunderink RG, Leeper Jr KV, Meduri GU, Steere EL, Glen Mayhall C: Epidemiology of ventilator-acquired pneumonia based on protected bronchoscopic sampling. Am J Respir Crit Care Med. 1998;158:1839-1847

S49. Georges H, Leroy O, Guery B, Alfandari S, Beaucaire G: Predisposing factors for nosocomial pneumonia in patients receiving mechanical ventilation and requiring tracheotomy. Chest. 2000;118:767–774.

S50. Giamarellos-Bourboulis EJ, Bengmark S, Kannellakopoulou K, Kotzampassi K: Pro- and synbiotics to control inflammation and infection in patients with multiple injuries. *J Trauma* 2009;67:815-821.

S51. Giard M, Lepape A, Allaouchiche B, Guerin C, Lehot JJ, Robert MO, Vanhems P: Early-and late-onset ventilator-associated pneumonia acquired in the intensive care unit: comparison of risk factors. *J Crit Care* 2008, 23:27-33.

S52. Gruson D, Hilbert G, Vargas F, Valentino R, Bebear C, Allery A, Bebear C, Gbikpi-Benissan GE, Cardinaud JP: Rotation and restricted use of antibiotics in a medical intensive care unit: impact on the incidence of ventilator-associated pneumonia caused by antibiotic-resistant gram-negative bacteria. Am J Respir Crit Care Med. 2000, 162(3):837-43.

S53. Gruson D, Hilbert G, Vargas F, Valentino R, Bui N, Pereyre S, Bebear C, Bebear CM, Gbikpi-Benissan G: Strategy of antibiotic rotation: long-term effect on incidence and susceptibilities of Gram-negative bacilli responsible for ventilator-associated pneumonia. Crit Care Med. 2003;31:1908-1914.

S54. Guérin C, Girard R, Chemorin C, De Varax R, Fournier G: Facial mask noninvasive mechanical ventilation reduces the incidence of nosocomial pneumonia. Intens care Med. 1997;23(10):1024-32.

S55. Gursel G, Aydogdu M, Nadir Ozis T, Tasyurek S. Comparison of the value of initial and serial endotracheal aspirate surveillance cultures in predicting the causative pathogen of ventilator-associated pneumonia. Scand J Infect Dis. 2010, 42:341-346.

S56. Heyland DK, Cook DJ, Schoenfeld PS, Frietag A, Varon J, Wood G: The effect of acidified enteral feeds on gastric colonization in critically ill patients: results of a multicenter randomized trial. Canadian Critical Care Trials Group. Crit Care Med. 1999;27:2399-2406

S57. Holzapfel L, Chevret S, Madinier G, Ohen F, Demingeon G, Coupry A, Chaudet M: Influence of long-term oro- or nasotracheal intubation on nosocomial maxillary sinusitis and pneumonia: results of a prospective, randomized, clinical trial. Crit Care Med. 1993;21:1132-1138

S58. Huang SS, Septimus E, Kleinman K, Moody J, Hickok J, Avery TR, Lankiewicz J, Gombosev A, Terpstra L, Hartford F, Hayden MK. Targeted versus universal decolonization to prevent ICU infection. *N Engl J Med*. 2013;368(24):2255-65.

S59. Hugonnet S, Uçkay I, Pittet D Staffing level: a determinant of late-onset ventilator-associated pneumonia. Crit Care. 2007;11(4):R80

S60. Hyllienmark P, Gardlund B, Persson JO, Ekdahl K. Nosocomial pneumonia in the ICU: a prospective cohort study. Scand J Infect Dis. 2007;39:676-82.

S61. Hyllienmark P, Brattström O, Larsson E, Martling CR, Petersson J, Oldner A: High incidence of post-injury pneumonia in intensive care-treated trauma patients. Acta Anaesthesiologica Scandinavica. 2013;57(7):848-54.

S62. Ibáñez J, Peñafiel A, Marsé P, Jordá R, Raurich JM, Mata F: Incidence of gastroesophageal reflux and aspiration in mechanically ventilated patients using small-bore nasogastric tubes. J Parenteral and Enteral Nutrition. 2000;24(2):103-6.

S63. Ibrahim EH, Ward S, Sherman G, Kollef MH: A comparative analysis of patients with early-onset vs late-onset nosocomial pneumonia in the ICU setting. Chest. 2000;117:1434-1442

S64. Ibrahim EH, Sherman G, Ward S, Fraser VJ, Kollef MH. The influence of inadequate antimicrobial treatment of bloodstream infections on patient outcomes in the ICU setting. Chest. 2000;118(1):146-55.

S65. Ibrahim EH, Tracy L, Hill C, et al. The occurrence of ventilator-associated pneumonia in a community hospital: risk factors and clinical outcomes. *Chest* 2001;120:555-61.

S66. Jacobs S, Chang RW, Lee B, Bartlett FW: Continuous enteral feeding: a major cause of pneumonia among ventilated intensive care unit patients. *JPEN J Parenter Enteral Nutr* 1990;14:353-6.

S67. Jaillette E, Nseir S: Relationship between inhaled β2-agonists and ventilator-associated pneumonia: A cohort study. Critical Care Med. 2011;39(4):725-30.

S68. Jensen JUS, Hein L, Lundgren B, Bestle MH, Mohr T, Andersen MH, Invasive *Candida* Infections and the Harm From Antibacterial Drugs in Critically Ill Patients: Data From a Randomized, Controlled Trial to Determine the Role of Ciprofloxacin, Piperacillin-Tazobactam, Meropenem, and Cefuroxime. *Crit Care Medicine* 2015;43(3):594-602.

S69. Jiménez P, Torres A, Rodríguez-Roisin R, de la Bellacasa JP, Aznar R, Gatell JM,: Incidence and etiology of pneumonia acquired during mechanical ventilation. Crit Care Med. 1989;17:882-5.

S70. Kallel H, Houcke S, Resiere D, Roy M, Mayence C, Mathien C, Mootien J, Demar M, Hommel D, Djossou F. Epidemiology and Prognosis of Intensive Care Unit–Acquired Bloodstream Infection. American J Trop med & Hygiene. 2020;103(1):508.

S71. Kautzky S, Staudinger T, Presterl E. Invasive Candida infections in patients of a medical intensive care unit. Wiener klinische Wochenschrift. 2015;127(3-4):132-42.

S72. Ko HK, Yu WK, Lien TC, Wang JH, Slutsky AS, Zhang H, Kou YR. Intensive care unit-acquired bacteremia in mechanically ventilated patients: clinical features and outcomes. *PloS one*. 2013;8(12):e83298.

S73. Kollef MH Ventilator-associated pneumonia. A multivariate analysis. JAMA 1993;270:1965-70.

S74. Kollef MH, Silver P, Murphy DM, Trovillion E: The effect of late-onset ventilator-associated pneumonia in determining patient mortality. Chest. 1995;108: 1655-62.

S75. Kollef MH, Shapiro SD, Fraser VJ, Silver P, Murphy DM, Trovillion E, Hearns ML, Richards RD, Cracchilo L, Hossin L: Mechanical ventilation with or without 7-day circuit changes. A randomized controlled trial. Ann Intern Med.1995;123:168–174

S76. Kollef MH, Von Harz B, Prentice D, Shapiro SD, Silver P, John RS, Trovillion E: Patient transport from intensive care increases the risk of developing ventilator-associated pneumonia. Chest. 1997;112(3):765-773.

S77. Kollef MH, Vlasnik JO, Sharpless L, Pasque C, Murphy D, Fraser V. Scheduled change of antibiotic classes: a strategy to decrease the incidence of ventilator-associated pneumonia. Am J Resp Crit Care Med. 1997;156(4):1040-8.

S78. Koss WG, Khalili TM, Lemus JF, Chelly MM, Margulies DR, Shabot MM: Nosocomial pneumonia is not prevented by protective contact isolation in the surgical intensive care unit. Am Surg. 2001;67:1140-4.

S79. Kunac A, Sifri ZC, Mohr AM, Horng H, Lavery RF, Livingston DH: Bacteremia and Ventilator-Associated Pneumonia: A Marker for Contemporaneous Extra-Pulmonic Infection. Surg Infect. 2014;15:77-83.

S80. Laggner AN, Lenz K, Base W, Druml W, Schneeweiss B, Grimm G: Prevention of upper gastrointestinal bleeding in long-term ventilated patients. Sucralfate versus ranitidine. Am J Med. 1989;86:81-4.

S81. Lambert ML, Suetens C, Savey A, Palomar M, Hiesmayr M, Morales I, Agodi A, Frank U, Mertens K, Schumacher M, Wolkewitz M. Clinical outcomes of health-care-associated infections and antimicrobial resistance in patients admitted to European intensive-care units: a cohort study. Lancet Infect Dis. 2011;11(1):30-8.

S82. Laupland KB, Zygun DA, Davies HD, Church DL, Louie TJ, Doig CJ Population-based assessment of intensive care unit-acquired bloodstream infections in adults: incidence, risk factors, and associated mortality rate. *Crit Care Med* 2002;30:2462-2467.

S83. Laupland KB, Kirkpatrick AW, Church DL, Ross T, Gregson DB Intensive-care-unit-acquired bloodstream infections in a regional critically ill population. *J Hosp Infect* 2004;58(2): 137-145.

S84. León C, Ruiz-Santana S, Saavedra P, Almirante B, Nolla-Salas J, Álvarez-Lerma F, Garnacho-Montero J, León MÁ. A bedside scoring system ("Candida score") for early antifungal treatment in nonneutropenic critically ill patients with Candida colonization. Crit Care Med. 2006;34(3):730-7.

S85. León C, Ruiz-Santana S, Saavedra P, Galván B, Blanco A, Castro C, Balasini C, Utande-Vázquez A, de Molina FJ, Blasco-Navalproto MA, López MJ. Usefulness of the "Candida score" for discriminating between Candida colonization and invasive candidiasis in non-neutropenic critically ill patients: a prospective multicenter study. Crit Care Med. 2009;37(5):1624-33.

S86. León C, Ruiz-Santana S, Saavedra P, Castro C, Loza A, Zakariya I, Úbeda A, Parra M, Macías D, Tomás JI, Rezusta A. Contribution of Candida biomarkers and DNA detection for the diagnosis of invasive candidiasis in ICU patients with severe abdominal conditions. Crit Care. 2016;20(1):149.

S87. Lepelletier D, Roquilly A, Mahe PJ, Loutrel O, Champin P, Corvec S, Naux E, Pinaud M, Lejus C,. Retrospective analysis of the risk factors and pathogens associated with early-onset ventilator-associated pneumonia in surgical-ICU head-trauma patients. J Neurosurg Anesthesiol. 2010;22(1):32-7.

S88. Li Z, Jiang C, Dong D, Zhang L, Tian Y, Ni Q, Mao E, Peng Y. The correlation between Candida colonization of distinct body sites and invasive candidiasis in emergency intensive care units: statistical and molecular biological analysis. Mycopathologia. 2016;181(7-8):475-84.

S89. Luna CM, Blanzaco D, Niederman MS, et al. Resolution of ventilator-associated pneumonia: prospective evaluation of the clinical pulmonary infection score as an early clinical predictor of outcome. Crit Care Med. 2003;31:676-682.

S90. Luyt CE, Guérin V, Combes A, Trouillet JL, Ayed SB, Bernard M, Gibert C, Chastre J: Procalcitonin kinetics as a prognostic marker of ventilator-associated pneumonia. Am J Respir Crit Care Med. 2005;171:48-53.

S91. Magnason S, Kristinsson KG, Stefansson T, Erlendsdottir H, Jonsdottir K, Kristjansson M, Gudmundsson S: Risk factors and outcome in ICU-acquired infections. Acta Anaesthesiol Scand. 2008;52:1238-1245

S92. Mahul P, Auboyer C, Jospe R, Ros A, Guerin C, el Khouri Z, Galliez M, Dumont A, Gaudin O: Prevention of nosocomial pneumonia in intubated patients respective role of mechanical subglottic secretions drainage and stress ulcer prophylaxis. Intensive Care Med. 1992;18:20-25

S93. Makris D, Manoulakas E, Komnos A, Papakrivou E, Tzovaras N, Hovas A, Zintzaras E, Zakynthinos E. Effect of pravastatin on the frequency of ventilator-associated pneumonia and on intensive care unit mortality: open-label, randomized study. Crit care med. 2011;39(11):2440-6.

S94. Markowicz P, Wolff M, Djedaini K, Cohen Y, Chastre J, Delclaux C: Multicenter prospective study of ventilator-associated pneumonia during acute respiratory distress syndrome. Incidence, prognosis, and risk factors. ARDS Study Group. Am J Respir Crit Care Med. 2000;161:1942-8.

S95. Memish ZA, Cunningham G, Oni GA, et al. The incidence and risk factors of ventilator-associated pneumonia in a Riyadh hospital. Infect Control Hosp Epidemiol. 2000;21:271-3.

S96. Michel F, Franceschini B, Berger P, Arnal JM, Gainnier M, Sainty JM, Papazian L. Early antibiotic treatment for BAL-confirmed ventilator-associated pneumonia: a role for routine endotracheal aspirate cultures. Chest. 2005;127(2):589-97.

S97. Mitsogianni M, Vasileiadis I, Parisi M, Tzanis G, Kampisiouli E, Psaroudaki Z, Perivolioti E, Fountoulis K, Routsi C, Nanas S, Tsiodras S. A Multifaceted Intervention Program to Prevent Bloodstream Infection in an IntensiveCare Unit. *Health Science J.* 2016;10(2):1.

S98. Moine P, Timsit JF, De Lassence A, Troché G, Fosse JP, Alberti C, Cohen Y: Mortality associated with late-onset pneumonia in the intensive care unit: results of a multi-center cohort study. Intensive Care Med. 2002;28:154-163

S99. Montecalvo MA, Steger KA, Farber HW: Nutritional outcome and pneumonia in critical care patients randomized to gastric versus jejunal tube feedings. The Critical Care Research Team. *Crit Care Med* 1992, 20:1377-1387.

S100. Myny D, Depuydt P, Colardyn F, Blot S: Ventilator-associated pneumonia in a tertiary care ICU analysis of risk factors for acquisition and mortality. Acta Clin Belg. 2005;60:114-121.

S101. Nguile-Makao M, Zahar JR, Français A, Tabah A, Garrouste-Orgeas M, Allaouchiche B, Goldgran-Toledano D, Azoulay E, Adrie C, Jamali S, Clec'h C. Attributable mortality of ventilator-associated pneumonia: respective impact of main characteristics at ICU admission and VAP onset using conditional logistic regression and multi-state models. Intens care med. 2010;36(5):781-9.

S102. Nielsen SL, Røder B, Magnussen P, Engquist A, Frimodt-møller N. Nosocomial pneumonia in an intensive care unit in a Danish university hospital: incidence, mortality and etiology. Scand J Infect Dis. 1992;24:65-70.

S103. Nseir S, Di Pompeo C, Soubrier S, Cavestri B, Jozefowicz E, Saulnier F, Durocher A: Impact of ventilator-associated pneumonia on outcome in patients with COPD. Chest. 2005;128(3):1650-1656.

S104. Nseir S, Jozefowicz E, Cavestri B, Sendid B, Di Pompeo C, Dewavrin F, Durocher A. Impact of antifungal treatment on Candida–Pseudomonas interaction: a preliminary retrospective case–control study. *Intensive Care Med* 2007;33(1):137-142.

S105. Orsi GB, Giuliano S, Franchi C, Ciorba V, Protano C, Giordano A, Rocco M, Venditti M. Changed epidemiology of ICU acquired bloodstream infections over 12 years in an Italian teaching hospital. *Minerva Anestesiol.* 2015;81(9):980-8.

S106. Osmon S, Warren D, Seiler SM, Shannon W, Fraser VJ, Kollef MH: The influence of infection on hospital mortality for patients requiring >48 h of intensive care. *Chest* 2003, 124:1021-1029.

S107. [OUTCOMEREA] Saied WI, Mourvillier B, Cohen Y, Ruckly S, Reignier J, Marcotte G, Siami S, Bouadma L, et al on behalf of the OUTCOMEREA study group. A comparison of the mortality risk associated with ventilator-acquired bacterial pneumonia and nonventilator ICU-acquired bacterial pneumonia. Crit care med. 2019;47:345-52.

S108. Papazian L, Bregeon F, Thirion X, Gregoire R, Saux P, Denis JP, Perin G, Charrel J, Dumon JF, Affray JP, Gouin F: Effect of ventilator-associated pneumonia on mortality and morbidity. Am J Respir Crit Care Med. 1996;154:91-7.

S109. Petri MG, König J, Moecke HP, Gramm HJ, Barkow H, Kujath P, Dennhart R, Lode H. Epidemiology of invasive mycosis in ICU patients: a prospective multicenter study in 435 non-neutropenic patients. *Intensive Care Med* 1997;23(3):317-325.

S110. Potgieter PD, Linton DM, Oliver S, Forder AA: Nosocomial infections in a respiratory intensive care unit. Crit Care Med. 1987;15:495-498

S111. Prowle JR, Echeverri JE, Ligabo EV, Sherry N, Taori GC, Crozier TM, Bellomo R. Acquired bloodstream infection in the intensive care unit: incidence and attributable mortality. Crit Care 2011;15(2):R100.

S112. Ramirez P, Lopez-Ferraz C, Gordon M, Gimeno A, Villarreal E, Ruiz J, Menendez R, Torres A. From starting mechanical ventilation to ventilator-associated pneumonia, choosing the right moment to start antibiotic treatment. *Crit Care* 2016;20(1):169.

S113. Rello J, Quintana E, Ausina V, Castella J, Luquin M, Net A, Prats G: Incidence, etiology, and outcome of nosocomial pneumonia in mechanically ventilated patients. Chest. 1991;100:439-444

S114. Rello J, Ausina V, Castella J, et al Nosocomial respiratory tract infections in multiple trauma patients. Influence of level of consciousness with implications for therapy. Chest 1992;102:525-529

S115. Rello J, Ricart M, Mirelis B, Quintana E, Gurgui M, Net A, Prats, G: Nosocomial bacteremia in a medical-surgical intensive care unit: epidemiologic characteristics and factors influencing mortality in 111 episodes. *Intensive Care Med* 1994;20:94-98.

S116. Rello J, Ausina V, Ricart M, Puzo C, Quintana E, Net A, Prats G. Risk factors for infection by *Pseudomonas aeruginosa* in patients with ventilator-associated pneumonia. *Intens Care Med*. 1994;20(3):193-8.

S117. Rello J, Ollendorf DA, Oster G, et al. Epidemiology and outcomes of ventilator-associated pneumonia in a large US database. Chest 2002;122:2115-2121

S118. Rello J, Lorente C, Diaz E, et al. Incidence, etiology, and outcome of nosocomial pneumonia in ICU patients requiring percutaneous tracheotomy for mechanical ventilation. *Chest* 2003;124:2239-43.

S119. Resende MM, Monteiro SG, Callegari B, Figueiredo PM, Monteiro CR, Monteiro-Neto V. Epidemiology and outcomes of ventilator-associated pneumonia in northern Brazil: an analytical descriptive prospective cohort study. BMC infect Dis 2013;13(1): 119

S120. Reusser P, Zimmerli W, Scheidegger D, Marbet GA, Buser M, Gyr K: Role of gastric colonization in nosocomial infections and endotoxemia: a prospective study in neurosurgical patients on mechanical ventilation. J Infect Dis. 1989;160:414-421

S121. Rincón-Ferrari MD, Flores-Cordero JM, Leal-Noval SR, Murillo-Cabezas F, Cayuelas A, Muñoz-Sánchez MA, Sánchez-Olmedo JI: Impact of ventilator-associated pneumonia in patients with severe head injury. J Trauma Acute Care Surg. 2004;57(6):1234-40.

S122. Rodrigues PM, Neto C, Santos LR, Knibel MF. Ventilator-associated pneumonia: epidemiology and impact on the clinical evolution of ICU patients. Jornal brasileiro de pneumologia. 2009 Nov;35(11):1084-91.

S123. Rodriguez JL, Gibbons KJ, Bitzer LG, Dechert RE, Steinberg SM, Flint LM: Pneumonia: incidence, risk factors, and outcome in injured patients. J Trauma. 1991;31: 907-12.

S124. Ruiz-Santana S, Garcia Jimenez A, Esteban A, et al. ICU pneumonias: a multi-institutional study. *Crit Care Med*. 1987;15:930-932.

S125. Salata RA, Lederman MM, Shlaes DM, Jacobs MR, Eckstein E, Tweardy D, Toossi Z, Chmielewski R, Marino J, King CH: Diagnosis of nosocomial pneumonia in intubated, intensive care unit patients. Am Rev Respir Dis. 1987;135:426-432

S126. Shahin J, Bielinski M, Guichon C, Flemming C, Kristof AS Suspected ventilator-associated respiratory infection in severely ill patients: a prospective observational study. Crit Care 2013;17: R251

S127. Sofianou DC, Constandinidis TC, Yannacou M, Anastasiou H, Sofianos E: Analysis of risk factors for ventilator-associated pneumonia in a multidisciplinary intensive care unit. *Eur J Clin Microbiol Infect Dis* 2000, 19:460-463.

S128. Stéphan F, Mabrouk N, Decailliot F, Delclaux C, Legrand P: Ventilator-associated pneumonia leading to acute lung injury after trauma: importance of *Haemophilus influenzae*. Anesthesiology. 2006;104: 235-41.

S129. Tan X, Zhu S, Yan D, Chen W, Chen R, Zou J, Yan J, Zhang X, Farmakiotis D, Mylonakis E. Candida spp. airway colonization: A potential risk factor for Acinetobacter baumannii ventilator-associated pneumonia. Med Mycol. 2016: 54; 557-66.

S130. Tejada Artigas AT, Dronda SB, Vallés EC, Marco JM, Usón MC, Figueras P, Suarez FJ, Hernandez A: Risk factors for nosocomial pneumonia in critically ill trauma patients. Crit Care Med. 2001;29:304-9.

S131. Thompson DS. Estimates of the rate of acquisition of bacteraemia and associated excess mortality in a general intensive care unit: a 10 year study. *J Hosp Infect*. 2008;69(1):56-61.

S132. Timsit JF, Chevret S, Valcke J, Misset B, Renaud B, Goldstein FW, Vaury P, Carlet J: Mortality of nosocomial pneumonia in ventilated patients: influence of diagnostic tools. Am J Respir Crit Care Med. 1996;154:116-23.

S133. Torres A, Aznar R, Gatell JM, Jiménez P, González J, Ferrer A, Celis R, Rodriguez-Roisin R: Incidence, risk, and prognosis factors of nosocomial pneumonia in mechanically ventilated patients. Am Rev Respir Dis. 1990;142:523-8.

S134. Trouillet JL, Chastre J, Vuagnat A, Joly-Guillou ML, Combaux D, Dombret MC, Gibert C: Ventilator-associated pneumonia caused by potentially drug-resistant bacteria. Am J Respir Crit Care Med. 1998;157(2):531-9.

S135. Urli T, Perone G, Acquarolo A, Zappa S, Antonini B, Ciani A: Surveillance of infections acquired in intensive care: usefulness in clinical practice. *J Hosp Infect* 2002, 52:130-5.

S136. Valles J, Pobo A, Garcia-Esquirol O, Mariscal D, Real J, Fernández R. Excess ICU mortality attributable to ventilator-associated pneumonia: the role of early vs late onset. *Intensive care medicine*, 2007;33(8):1363-1368.

S137. Vanhems P, Bénet T, Voirin N, Januel JM, Lepape A, Allaouchiche B, Argaud L, Chassard D, Guérin C. Early-onset ventilator-associated pneumonia incidence in intensive care units: a surveillance-based study. BMC Infect Dis. 2011;11(1):236.

S138. Verhamme KM, De Coster W, De Roo L, De Beenhouwer H, Nollet G, Verbeke J, Demeyer I, Jordens P: Pathogens in early-onset and late-onset intensive care unit–acquired pneumonia. Infection Control Hospital Epidemiol. 2007;28(4):389-397.

S139. Violan JS, Sanchez-Ramirez C, Mujica AP, Cendrero JC, Fernandez JA, de Castro FR: Impact of nosocomial pneumonia on the outcome of mechanically-ventilated patients. Crit Care (Lond). 1998;2:19-23.

S140. Warren DK, Zack JE, Elward AM, Cox MJ, Fraser VJ. Nosocomial primary bloodstream infections in intensive care unit patients in a nonteaching community medical center: a 21-month prospective study. *Clin Infect Dis.* 2001;33(8):1329-35.

S141. Woske HJ, Röding T, Schulz I, Lode H: Ventilator-associated pneumonia in a surgical intensive care unit Epidemiology, etiology and comparison of three bronchoscopic methods for microbiological specimen sampling. Crit Care. 2001;5:167–173.

S142. Xie DS, Xiong W, Lai RP, Liu L, Gan XM, Wang XH, Nie SF. Ventilator-associated pneumonia in intensive care units in Hubei Province, China: a multicentre prospective cohort survey. J Hosp Infect 2011;78(4): 284-288

S143. Xie J, Li S, Xue M, Yang C, Huang Y, Chihade DB, Liu L, Yang Y, Qiu H. Early-and Late-Onset Bloodstream Infections in the Intensive Care Unit: A Retrospective 5-Year Study of Patients at a University Hospital in China. *J Infect Dis.* 16;221(Supplement_2):S184-92.

S144. Zahar JR, Nguile-Makao M, Français A, Schwebel C, Garrouste-Orgeas M, Goldgran-Toledano D, Azoulay E, Thuong M, Jamali S, Cohen Y, De Lassence A. Predicting the risk of documented ventilator-associated pneumonia for benchmarking: construction and validation of a score. Crit care med. 2009;37(9):2545-51.

S145. Acosta-Escribano J, Fernández-Vivas M, Carmona TG, Caturla-Such J, Garcia-Martinez M, Menendez-Mainer A, Sanchez-Payá J (2010) Gastric versus transpyloric feeding in severe traumatic brain injury: a prospective, randomized trial. Intensive Care Med 36:1532-1539

S146. Bonten MJ, Gaillard CA, Van der Geest S, Van Tiel FH, Beysens AJ, Smeets HG, Stobberingh EE: The role of intragastric acidity and stress ulcer prophylaxis on colonization and infection in mechanically ventilated ICU patients. A stratified, randomized, double-blind study of sucralfate versus antacids. Am J Respir Crit Care Med. 1995;152:1825-1834.

S147. Cook D, Guyatt G, Marshall J, et al A comparison of sucralfate and ranitidine for the prevention of upper gastrointestinal bleeding in patients requiring mechanical ventilation. Canadian Critical Care Trials Group. N Engl J Med 1998;338:791-797

S148. Daumal F, Colpart E, Manoury B, Mariani M, Daumal M. Changing heat and moisture exchangers every 48 hours does not increase the incidence of nosocomial pneumonia. Infection Control & Hospital Epidemiology. 1999;20(5):347-9.

S149. Djedaini K, Billiard M, Mier L, Le Bourdelles G, Brun P, Markowicz P, Estagnasie P, Coste F, Boussougant Y, Dreyfuss D: Changing heat and moisture exchangers every 48 hours rather than 24 hours does not affect their efficacy and the incidence of nosocomial pneumonia. Am J Respir Crit Care Med. 1995;152(5):1562-9.

S150. Drakulovic MB, Torres A, Bauer TT, Nicolas JM, Nogué S, Ferrer M: Supine body position as a risk factor for nosocomial pneumonia in mechanically ventilated patients: a randomised trial. Lancet. 1999;354(9193):1851-1858

S151. Dreyfuss D, Djedaini K, Weber P, Brun P, Lanore JJ, Rahmani J, Coste F: Prospective study of nosocomial pneumonia and of patient and circuit colonization during mechanical ventilation with circuit changes every 48 hours versus no change. Am Rev Respir Dis. 1991;143(4 Pt 1), 738-743.

S152. Dreyfuss D, Djedaïni K, Gros I, Mier L, Le Bourdellés G, Cohen Y, Estagnasié P, Coste F, Boussougant Y: Mechanical ventilation with heated humidifiers or heat and moisture exchangers: effects on patient colonization and incidence of nosocomial pneumonia. Am J Respir Crit Care Med. 1995;151:986-92.

S153. Driks MR, Craven DE, Celli BR, et al (1987) Nosocomial pneumonia in intubated patients given sucralfate as compared with antacids or histamine type 2 blockers. The role of gastric colonization. N Engl J Med 317:1376-1382

S154. Forestier C, Guelon D, Cluytens V, Guillart T, Sirot J, De champs C: Oral probiotic and prevention of *Pseudomonas aeruginosa* infections: a randomized, double-blind, placebo controlled pilot study in intensive care unit patients. *Crit Care* 2008;12:R69.

S155. Heyland DK, Cook DJ, Schoenfeld PS, Frietag A, Varon J, Wood G The effect of acidified enteral feeds on gastric colonization in critically ill patients: results of a multicenter randomized trial. Canadian Critical Care Trials Group. Crit Care Med 1999;27:2399-2406

S156. Holzapfel L, Chastang C, Demingeon G, Bohe J, Piralla B, Coupry A: A randomized study assessing the systematic search for maxillary sinusitis in nasotracheally mechanically ventilated patients. Influence of nosocomial maxillary sinusitis on the occurrence of ventilator-associated pneumonia. Am J Respir Crit Care Med. 1999;159:695-701

S157. Kappstein I, Schulgen G, Friedrich T, Hellinger P, Benzing A, Geiger K, Daschner FD. Incidence of pneumonia in mechanically ventilated patients treated with sucralfate or cimetidine as prophylaxis for stress bleeding: bacterial colonization of the stomach. Am J Med. 1991;91(2):S125-31

S158. Kirschenbaum L, Azzi E, Sfeir T, et al. Effect of continuous lateral rotational therapy on the prevalence of ventilator-associated pneumonia in patients requiring long-term ventilatory care *Crit Care Med* 2002;30:1983-6.

S159. Kirton OC, DeHaven B, Morgan J, et al. A prospective, randomized comparison of an in-line heat moisture exchange filter and heated wire humidifiers: rates of ventilator-associated early-onset (community-acquired) or late-onset (hospital-acquired) pneumonia and incidence of endotracheal tube occlusion. *Chest* 1997;112:1055-9.

S160. Knight DJ, Gardiner D, Banks A, Snape SE, Weston VC, Bengmark S, Girling KJ: Effect of synbiotic therapy on the incidence of ventilator associated pneumonia in critically ill patients: a randomised, double-blind, placebo-controlled trial. Intensive Care Med. 2009;35:854-861.

S161. Kollef MH, Afessa B, Anzueto A, Veremakis C, Kerr KM, Margolis BD, Schinner R: Silver-coated endotracheal tubes and incidence of ventilator-associated pneumonia: the NASCENT randomized trial. JAMA. 2008;300(7):805-813

S162. Lacherade JC, Auburtin M, Cerf C, Van de Louw A, et al. Impact of humidification systems on ventilator-associated pneumonia: a randomized multicenter trial. Am J Respir Crit Care Med. 2005;172:1276-82

S163. Lacherade JC, De Jonghe B, Guezennec P, Debbat K, Hayon J, Monsel A, Bastuji-Garin S: Intermittent subglottic secretion drainage and ventilator-associated pneumonia A multicenter trial. Am J Respir Crit Care Med. 2010;182:910-917.

S164. Launey Y, Nesseler N, Le Cousin A, Feuillet F, Garlantezec R, Mallédant Y, Seguin P: Effect of a fever control protocol-based strategy on ventilator-associated pneumonia in severely brain-injured patients. Crit Care. 2014;18(6):1.

S165. Lorente L, Lecuona M, Málaga J, Revert C, Mora ML, Sierra A: Bacterial filters in respiratory circuits: an unnecessary cost? Crit Care Med 2003;31:2126-2130

S166. Lorente L, Lecuona M, Galván R, Ramos MJ, Mora ML, Sierra A: Periodically changing ventilator circuits is not necessary to prevent ventilator-associated pneumonia when a heat and moisture exchanger is used. Infect Control Hosp Epidemiol. 2004;25:1077-1082

S167. Lorente L, Lecuona M, Martín MM, García C, Mora ML, Sierra A: Ventilator-associated pneumonia using a closed versus an open tracheal suction system. Crit Care Med. 2005;33:115-119

S168. Lorente L, Lecuona M, Jiménez A, Mora ML, Sierra A: Tracheal suction by closed system without daily change versus open system. Intensive Care Med. 2006;32:538-44.

S169. Lorente L, Lecuona M, Jimenez A, Mora ML, Sierra A: Ventilator-associated pneumonia using a heated humidifier or a heat and moisture exchanger: a randomized controlled trial [ISRCTN88724583]. Crit Care 2006;10:R116

S170. Lorente L, Lecuona M, Jimenez A, Mora ML, Sierra: Influence of an endotracheal tube with polyurethane cuff and subglottic secretion drainage on pneumonia. Am J Respir Crit Care Med. 2007;176:1079-1083

S171. Lorente L, Lecuona M, Jiménez A, Lorenzo L, Roca I, Cabrera J, Llanos C, Mora ML: Continuous endotracheal tube cuff pressure control system protects against ventilator-associated pneumonia. Crit Care. 2014;18(2):1.

S172. Manzano F, Fernandez-Mondejar E, Colmenero M, Poyatos ME, Rivera R, Machado J, Catalan I, Artigas A: Positive-end expiratory pressure reduces incidence of ventilator-associated pneumonia in nonhypoxemic patients. Crit Care Med: 2008;36(8):2225-31.

S173. Martin C, Perrin G, Gevaudan MJ, Saux P, Gouin F. Heat and moisture exchangers and vaporizing humidifiers in the intensive care unit. Chest. 1990;97(1):144-9.

S174. Morrow LE, Kollef MH, Casale TB: Probiotic prophylaxis of ventilator-associated pneumonia: a blinded, randomized, controlled trial. Am J Respir Crit Care Med. 2010;182:1058-1064

S175. Nseir S, Zerimech F, Fournier C, Lubret R, Ramon P, Durocher A, Balduyck M: Continuous control of tracheal cuff pressure and microaspiration of gastric contents in critically ill patients. Am J Respir Crit Care Med. 2011;184(9):1041-7.

S176. Pickworth KK, Falcone RE, Hoogeboom JE, et al Occurrence of nosocomial pneumonia in mechanically ventilated trauma patients: a comparison of sucralfate and ranitidine. Crit Care Med 1993;21:1856-1862

S177. Pneumatikos I, Konstantonis D, Tsagaris I, Theodorou V, Vretzakis G, Danielides V, Bouros D: Prevention of nosocomial maxillary sinusitis in the ICU: the effects of topically applied alpha-adrenergic agonists and corticosteroids. Intensive Care Med. 2006;32:532-537

S178. Prod'hom G, Leuenberger P, Koerfer J, Blum A, Chiolero R, Schaller MD, Perret C, Spinnler O, Blondel J, Siegrist H, Saghafi L: Nosocomial pneumonia in mechanically ventilated patients receiving antacid, ranitidine, or sucralfate as prophylaxis for stress ulcer. A randomized controlled trial. Ann Intern Med. 1994;120:653-62.

S179. Reignier J, Mercier E, Le Gouge A, et al: Effect of Not Monitoring Residual Gastric Volume on Risk of Ventilator-Associated Pneumonia in Adults Receiving Mechanical Ventilation and Early Enteral Feeding. A Randomized Controlled Trial. *JAMA* 2013, 309;249-256.

S180. Rumbak MJ, Truncale T, Newton MN, Adams B, Hazard P. A Prospective, Randomized Study Comparing Early Versus Delayed Percutaneous Tracheostomy In Critically Ill Medical Patients Requiring Prolonged Mechanical Ventilation. Chest. 2000;118(4):97S-8S.

S181. Ryan P, Dawson J, Teres D, Celoria G, Navab F: Nosocomial pneumonia during stress ulcer prophylaxis with cimetidine and sucralfate. Arch Surg. 1993;128(12):1353-7.

S182. Smulders K, van der Hoeven H, Weers-Pothoff I, Vandenbroucke-Grauls C A randomized clinical trial of intermittent subglottic secretion drainage in patients receiving mechanical ventilation. Chest 2002;121:858-862

S183. Staudinger T, Bojic A, Holzinger U, Meyer B, Rohwer M, Mallner F, Locker GJ Continuous lateral rotation therapy to prevent ventilator-associated pneumonia Crit Care Med 2010;38(2):486-490

S184. Thomachot L, Viviand X, Arnaud S, Boisson C, Martin CD: Comparing two heat and moisture exchangers, one hydrophobic and one hygroscopic, on humidifying efficacy and the rate of nosocomial pneumonia. Chest. 1998;114:1383-1389

S185. Thomachot L, Leone M, Razzouk K, Antonini F, Vialet R, Martin C: Do the components of heat and moisture exchanger filters affect humidifying efficacy and the incidence of nosocomial pneumonia? Crit Care Med. 1999;27:923–928

S186. Thomachot L, Leone M, Razzouk K, Antonini F, Vialet R, Martin C: Randomized Clinical Trial of Extended Use of a Hydrophobic Condenser Humidifier: 1 vs 7 Days. Crit Care Med. 2002;30:232-7

S187. Valencia M, Ferrer M, Farre R, Navajas D, Badia JR, Nicolas JM, Torres A: Automatic control of tracheal tube cuff pressure in ventilated patients in semirecumbent position: a randomized trial. Crit Care Med. 2007;35: 1543-9.

S188. Walaszek M, Gniadek A, Kolpa M, Wolak Z, Kosiarska A. The effect of subglottic secretion drainage on the incidence of ventilator associated pneumonia. *Biomed Pap Med Fac Univ Palacky Olomouc Czech Repub*. 2017;161(4):374-80.

S189. Zeng J, Wang CT, Zhang FS, Qi F, Wang SF, Ma S, Wu TJ, Tian H, Tian ZT, Zhang SL, Qu Y. Effect of probiotics on the incidence of ventilator-associated pneumonia in critically ill patients: a randomized controlled multicenter trial. Intens care med. 2016;42(6):1018-28.

S190. Bellissimo-Rodrigues WT, Menegueti MG, Gaspar GG, Nicolini EA, Auxiliadora-Martins M, Basile-Filho A, Bellissimo-Rodrigues F. Effectiveness of a Dental Care Intervention in the Prevention of Lower Respiratory Tract Nosocomial Infections among Intensive Care Patients: A Randomized Clinical Trial. Infect Control Hosp Epidemiol 2014, 35:1342-1348

S191. Bleasdale SC, Trick WE, Gonzalez IM, Lyles RD, Hayden MK, Weinstein RA. Effectiveness of chlorhexidine bathing to reduce catheter-associated bloodstream infections in medical intensive care unit patients. Arch intern med. 2007;167(19):2073-9.

S192. Ćabov T, Macan D, Husedžinović I, Škrlin-Šubić J, Bošnjak D, Šestan-Crnek S, Perić B, Kovač Z, Golubović V. The impact of oral health and 0.2% chlorhexidine oral gel on the prevalence of nosocomial infections in surgical intensive-care patients: a randomized placebo-controlled study. Wiener klinische Wochenschrift. 2010;122(13-14):397-404.

S193. Caruso P, Denari S, Ruiz SA, Demarzo SE, Deheinzelin D. Saline instillation before tracheal suctioning decreases the incidence of ventilator-associated pneumonia. Crit Care Med 2009, 37:32-38.

S194. Climo MW, Yokoe DS, Warren DK et al. Effect of daily chlorhexidine bathing on hospital-acquired infection. *N Engl J Med* 2013; 368: 533–542.

S195. Fourrier FE, Cau-Pottier H, Boutigny M, Roussel-Delvallez M, Jourdain, Chopin C: Effects of dental plaque antiseptic decontamination on bacterial colonization and nosocomial infections in critically ill patients. Intensive Care Med. 2000;26:1239-1247

S196. Fourrier F, Dubois D, Pronnier P, et al: Effect of gingival and dental plaque antiseptic decontamination on nosocomial infections acquired in the intensive care unit a double-blind placebo-controlled multicenter study. Crit Care Med. 2005;33:1728-1735

S197. Koeman M, van der Ven AJ, Hak E, et al. Oral decontamination with chlorhexidine reduces the incidence of ventilator-associated pneumonia. Am J Respir Crit Care Med 2006;173:1348-1355

S198. Kollef M, Pittet D, Sanchez Garcia M, et al. A randomized double-blind trial of iseganan in prevention of ventilator-associated pneumonia. *Am J Respir Crit Care Med* 2006: 173:91-7.

S199. Lorente L, Lecuona M, Jiménez A, Palmero S, Pastor E, Lafuente N, Ramos MJ, Mora ML, Sierra A: Ventilator-associated pneumonia with or without toothbrushing a randomized controlled trial. *Eur J Clin Microbiol Infect Dis.* 2012;31:1-9

S200. Milstone AM, Elward A, Song X, Zerr DM, Orscheln R, Speck K, Obeng D, Reich NG, Coffin SE, Perl TM, Pediatric SCRUB Trial Study Group. Daily chlorhexidine bathing to reduce bacteraemia in critically ill children: a multicentre, cluster-randomised, crossover trial. *The Lancet*. 2013;381(9872):1099-106.

S201. Mori H, Hirasawa H, Oda S, Shiga H, Matsuda K, Nakamura M: Oral care reduces incidence of ventilator-associated pneumonia in ICU populations. Intensive Care Med, 2006, 32(2), 230-236.

S202. Noto MJ, Domenico HJ, Byrne DW, et al. Chlorhexidine bathing and health care–associated infections: a randomized clinical trial. *JAMA*. 2015;313(4):369-78.

S203. Seguin P, Tanguy M , Laviolle B, Tirel O, Malledant Y: Effect of oropharyngeal decontamination by povidone-iodine on ventilator-associated pneumonia in patients with head trauma. *Crit Care Med* 2006, 34:1514-1519.

S204. Seguin P, Laviolle B, Dahyot-Fizelier C, Dumont R, Veber B, Gergaud S, Asehnoune K, Mimoz O, Donnio PY, Bellissant E, Malledant Y. Effect of oropharyngeal povidone-iodine preventive oral care on ventilator-associated pneumonia in severely brain-injured or cerebral hemorrhage patients: a multicenter, randomized controlled trial. Crit care med. 2014;42(1):1-8.

S205. Swan JT, Ashton CM, Bui LN, Pham VP, Shirkey BA, Blackshear JE, Bersamin JB, Pomer RM, Johnson ML, Magtoto AD, Butler MO. Effect of chlorhexidine bathing every other day on prevention of hospital-acquired infections in the surgical ICU: a single-center, randomized controlled trial. Crit Care Med. 2016;44(10):1822-32.

S206. Wittekamp BH, Plantinga NL, Cooper BS, et al. Decontamination strategies and bloodstream infections with antibiotic-resistant microorganisms in ventilated patients: a randomized clinical trial. *JAMA*. 2018.

S207. Bergmans DC, Bonten MJ, Gaillard CA, et al Prevention of ventilator-associated pneumonia by oral decontamination: a prospective, randomized, double-blind, placebo-controlled study. Am J Respir Crit Care Med 2001;164:382-388

S208. Bonten MJ, Gaillard CA, Johanson Jr WG, Van Tiel FH, Smeets HG, Van Der Geest S, Stobberingh EE. Colonization in patients receiving and not receiving topical antimicrobial prophylaxis. *Am J Respir Crit Care Med* 1994;150(5):1332-1340.

S209. Camus C, Salomon S, Bouchigny C, et al. Short-term decline in all-cause acquired infections with the routine use of a decontamination regimen combining topical polymyxin, tobramycin, and amphotericin B with mupirocin and chlorhexidine in the ICU: a single-center experience. *Crit Care Med.* 2014;42(5):1121-30.

S210. De la court Jara R, Sigaloff KC, Groot T, van der Spoel JI, Schade RP. Reducing the dosing frequency of selective digestive tract decontamination to three times daily provides effective decontamination of Gram-negative bacteria. *Eur J Clin Microbiol Infect Dis*. 2021 1:1-8.

S211. de Smet AMGA, Kluytmans JAJW, Cooper BS, et al: Decontamination of the digestive tract and oropharynx in ICU patients. *N Engl J Med* 2009, 360:20–31.

S212. Godard J, Guillaume C, Reverdy ME, Bachmann P, Bui-Xuan B, Nageotte A, Motin J: Intestinal decontamination in a polyvalent ICU. A double-blind study. *Intensive Care Med* 1990, 16:307-311.

S213. Gorensek MJ, Carey WD, Washington 2nd JA, Vogt DP, Broughan TA, Westveer MK. Selective bowel decontamination with quinolones and nystatin reduces gram-negative and fungal infections in orthotopic liver transplant recipients. Cleveland Clin J Med. 1993;60(2):139-44.

S214. Hartenauer UB, Thülig B, Lawin P, Fegeler W. Infection surveillance and selective decontamination of the digestive tract (SDD) in critically ill patients—results of a controlled study. *Infection.* 1990;18(1):S22-30.

S215. Hjortrup A, Rasmussen A, Hansen BA, Hoiby N, Heslet L, Moesgaard F, Kirkegaard P (1997) Early bacterial and fungal infections in liver transplantation after oral selective bowel decontamination. *Transplantation proceedings* 29:3106-3110

S216. Konrad F, Schwalbe B, Heeg K, et al. [Frequency of colonization and pneumonia and development of resistance in long-term ventilated intensive-care patients subjected to selective decontamination of the digestive tract]. *Anaesthesist* 1989;38:99-109.

S217. Landelle C, Boyer VN, Abbas M, Genevois E, Abidi N, Naimo S, Raulais R, Bouchoud L, Boroli F, Terrisse H, Bosson JL. Impact of a multifaceted prevention program on ventilator-associated pneumonia including selective oropharyngeal decontamination. *Intensive Care Med* 2018;44:1777-86.

S218. Ledingham I, Eastaway A, Mckay I, Alcock S, Mcdonald J, Ramsay G: Triple regimen of selective decontamination of the digestive tract, systemic cefotaxime, and microbiological surveillance for prevention of acquired infection in intensive care. *Lancet* 1988, 1:785-90.

S219. Leone M, Bourgoin A, Giuly E, et al. Influence on outcome of ventilator-associated pneumonia in multiple trauma patients with head trauma treated with selected digestive decontamination. *Crit Care Med* 2002; 30:1741-6.

S220. Mathieu C, Abbate R, Meresse Z, Hammad E, Duclos G, Antonini F, Cassir N, Schouten J, Zieleskiewicz L, Leone M. Decreased duration of intravenous cephalosporins in intensive care unit patients with selective digestive decontamination: a retrospective before-and-after study. *Eur J Clin Microbiol Infect Dis*. 2020;39(11):2115-20.

S221. Nardi G, Valentinis U, Bartaletti R, Bello A, De AM, Muzzi R, Giordano F, Troncon MG. Effectiveness of topical selective decontamination, without systemic antibiotic prophylaxis, in prevention of pulmonary infection in intensive care. *Minerva anestesiologica.* 1990;56(1-2):19-26.

S222. Nardi G, Di Silvestre A, De Monte A, Massarutti D, Proietti A, Troncon MG, Zussino M: Reduction in gram-positive pneumonia and antibiotic consumption following the use of a SDD protocol including nasal and oral mupirocin. Eur J Emerg Med 2001;8:203-214

S223. Ong DS, Bonten MJ, Safdari K, Spitoni C, Frencken JF, Witteveen E, Horn J, Klein Klouwenberg PM, Cremer OL, MARS consortium, de Beer FM. Epidemiology, management, and risk-adjusted mortality of ICU-acquired enterococcal bacteremia. *Clin Infect Dis* 2015;61(9):1413-20.

S224. Oostdijk EAN, Kesecioglu J, Schultz MJ, et al. Notice of Retraction and Replacement: Oostdijk et al. Effects of Decontamination of the Oropharynx and Intestinal Tract on Antibiotic Resistance in ICUs: A Randomized Clinical Trial. *JAMA*. 2014;312(14):1429-1437. *JAMA* 2017

S225. Rouby JJ, Poete P, de Lassale EM, Nicolas MH, Bodin L, Jarlier V, Korinek AM, Viars P. Prevention of Gram negative noscomial bronchopneumonia by intratracheal colistin in critically ill patients. *Intensive Care Med*. 1994;20(3):187-92.

S226. Silvestri L, Bragadin CM, Milanese M, Gregori D, Consales C, Gullo A, Van Saene HK. Are most ICU infections really nosocomial? A prospective observational cohort study in mechanically ventilated patients. *J Hosp Infect.* 1999;42(2):125-33.

S227. Steffen R, Reinhartz O, Blumhardt G, Bechstein WO, Raakow R, Langrehr JM, Rossaint R, Slama K, Neuhaus P. Bacterial and fungal colonization and infections using oral selective bowel decontamination in orthotopic liver transplantations. *Transpl Inter.* 1994;7(2):101-8.

S228. Stoutenbeek CP, Van Saene HK, Miranda DR, Zandstra DF. The effect of selective decontamination of the digestive tract on colonisation and infection rate in multiple trauma patients. Intens Care Med. 1984;10(4):185-92.

S229. Stoutenbeek CP, van Saene HK, Miranda DR, Zandstra DF, Langrehr D; The effect of oropharyngeal decontamination using topical nonabsorbable antibiotics on the incidence of nosocomial respiratory tract infections in multiple trauma patients. J Trauma 1987;27:357-364

S230. The SuDDICU Investigators for the Australian and New Zealand Intensive Care Society Clinical Trials Group. Effect of selective decontamination of the digestive tract on hospital mortality in critically ill patients receiving mechanical ventilation: a randomized clinical trial. JAMA. Published October 26, 2022. doi:10.1001/jama.2022.17927

S231. Vallés J, Peredo R, Burgueño MJ, de Freitas AP, Millán S, Espasa M, Martín-Loeches I, Ferrer R, Suarez D, Artigas A. Efficacy of single-dose antibiotic against early-onset pneumonia in comatose patients who are ventilated. *Chest.* 2013;143(5):1219-25.

S232. Veelo DP, Bulut T, Dongelmans DA, et al. The incidence and microbial spectrum of ventilator-associated pneumonia after tracheotomy in a selective decontamination of the digestive tract-setting. *J Infect* 2008; 56:20-6.

S233. Winter R, Humphreys H, Pick A, MacGowan AP, Willatts SM, Speller DC: A controlled trial of selective decontamination of the digestive tract in intensive care and its effect on nosocomial infection. J Antimicrob Chemother. 1992;30:73-87

S234. Abele-Horn M, Dauber A, Bauernfeind A, Russwurm W, Seyfarth-Metzger I, Gleich P, Ruckdeschel G: Decrease in nosocomial pneumonia in ventilated patients by selective oropharyngeal decontamination (SOD). Intensive Care Med. 1997;23:187-95.

S235. Acquarolo A, Urli T, Perone G, Giannotti C, Candiani A, Latronico N. Antibiotic prophylaxis of early onset pneumonia in critically ill comatose patients. A randomized study. Intensive Care Med. 2005; 31(4):510-6.

S236. Aerdts SJ, van Dalen R, Clasener HA, Festen J, van Lier HJ, Vollaard EJ: Antibiotic prophylaxis of respiratory tract infection in mechanically ventilated patients. A prospective, blinded, randomized trial of the effect of a novel regimen. Chest. 1991;100:783-791

S237. Bion JF, Badger I, Crosby HA, Hutchings P, Kong KL, Baker J, Hutton P, McMaster P, Buckels JA, Elliott TSJ: Selective decontamination of the digestive tract reduces gram-negative pulmonary colonization but not systemic endotoxemia in patients undergoing elective liver transplantation. Crit Care Med. 1994;22:40-49

S238. Blair P, Rowlands BJ, Lowry K, Webb H, Armstrong P, Smilie J Selective decontamination of the digestive tract: a stratified, randomized, prospective study in a mixed intensive care unit. Surgery 1991;110:303-309

S239. Blaise M, Pateron D, Trinchet JC, Levacher S, Beaugrand M, Pourriat JL. Systemic antibiotic therapy prevents bacterial infection in cirrhotic patients with gastrointestinal hemorrhage. Hepatology. 1994;20(1):34-8.

S240. Bouza E, Granda MJ, Hortal J, Barrio JM, Cercenado E, Muñoz P. Pre-emptive broad-spectrum treatment for ventilator-associated pneumonia in high-risk patients. *Intensive Care Med* 2013;39(9):1547-55.

S241. Camus C, Bellissant E, Sebille V, Perrotin D, Garo B, Legras A, Renault A, Le Corre P, Donnio PY, Gacouin A: Prevention of acquired infections in intubated patients with the combination of two decontamination regimens. *Crit Care Med* 2005, 33:307-314.

S242. Cerra FB, Maddaus MA, Dunn DL, Wells CL, Konstantinides NN, Lehmann SL, Mann HJ. Selective gut decontamination reduces nosocomial infections and length of stay but not mortality or organ failure in surgical intensive care unit patients. Arch Surg 1992;127:163-167.

S243. Cockerill FR, 3rd, Muller SR, Anhalt JP, et al. Prevention of infection in critically ill patients by selective decontamination of the digestive tract. *Ann Intern Med* 1992;117:545-53.

S244. de La Cal MA, Cerdá E, Garcıa-Hierro P, Van Saene HK. G ómez-Santos D, Negro E & Lorente JA. Survival benefit in critically ill burned patients receiving selective decontamination of the digestive tract: a randomized, placebocontrolled, double-blind trial. Ann Surg. 2005;241:424-30.

S245. Ferrer M, Torres A, Gonzalez J, Puig de la Bellacasa J, el-Ebiary M, Roca M, Gatell JM, Rodriguez-Roisin R: Utility of selective digestive decontamination in mechanically ventilated patients. Ann Intern Med. 1994;120:389-395

S246. Flaherty J, Nathan C, Kabins SA, Weinstein RA. Pilot trial of selective decontamination for prevention of bacterial infection in an intensive care unit. J Infect Dis 1990;162:1393-1397.

S247. François B, Cariou A, Clere-Jehl R, Dequin PF, Renon-Carron F, Daix T, Guitton C, Deye N, Legriel S, Plantefève G, Quenot JP. Prevention of early ventilator-associated pneumonia after cardiac arrest. *N Engl J Med*. 2019;381(19):1831-42.

S248. Garbino J, Pichard C, Pichna P, Pittet D, Lew D, Romand J (2004) Impact of enteral versus parenteral nutrition on the incidence of fungal infections: a retrospective study in ICU patients on mechanical ventilation with selective digestive decontamination. Clinical Nutrition 2004;23;705-710.

S249. Gaussorgues P, Salord M, Sirodot S, Tigaud S, Cagnin S, Gerard M, Robert D. Efficiency of selective decontamination of the digestive tract on the occurrence of nosocomial bacteremia in patients on mechanical ventilation receiving betamimetic therapy. Réan Soins Intens Méd Urg 1991;7:169-174.

S250. Georges B, Mazerolles M, Decun J-F, Rouge P, Pomies S, Cougot P Décontamination digestive sélective résultats d'une étude chez le polytraumatisé. *Réanimation Soins Intensifs Médecin d'Urgence* 1994;3:621-627

S251. Hammond JM, Potgieter PD, Saunders LG. Selective decontamination of the digestive tract in multiple trauma patients-Is there a role? Results of a prospective, double-blind, randomized trial. Crit Care Med. 1994;22(1):33-9.

S252. Jacobs S, Foweraker JE, Roberts SE: Effectiveness of selective decontamination of the digestive tract (SDD) in an ICU with a policy encouraging a low gastric pH. Clin Intensive Med. 1992;3:52-58

S253. Karvouniaris M, Makris D, Zygoulis P, Triantaris A, Xitsas S, Mantzarlis K, Petinaki E, Zakynthinos E. Nebulised colistin for ventilator-associated pneumonia prevention. Eur Resp J. 2015:46:1544-1547.

S254. Kerver AJ, Rommes JH, Mevissen-Verhage EA, et al Prevention of colonization and infection in critically ill patients: a prospective randomized study. Crit Care Med 1988;16:1087-1093.

S255. Korinek AM, Laisne MJ, Nicolas MH, Raskine L, Deroin V, Sanson-lepors MJ: Selective decontamination of the digestive tract in neurosurgical intensive care unit patients: a double-blind, randomized, placebo-controlled study. Crit Care Med. 1993;21:1466-73.

S256. Laggner AN, Tryba M, Georgopoulos A, Lenz K, Grimm G, Graninger W, Schneeweiss B, Druml W: Oropharyngeal decontamination with gentamicin for long-term ventilated patients on stress ulcer prophylaxis with sucralfate? *Wien Klin Wochenschr* 1994, 106:15-19.

S257. Langlois-Karaga A, Bues-Charbit M, Davignon A, Albanese J, Durbec O, Martin C, Morati N, Balansard G. Selective digestive decontamination in multiple trauma patients: cost and efficacy. *Pharmacy World and Science*. 1995;17(1):12-6.

S258. Mirtalaei N, Farazi A, Monfared ME, Jokar A. Efficacy of antibiotic prophylaxis against ventilator-associated pneumonia. J Hosp Infection. 2019;101(3):272-5.

S259. Palomar M, Alvarez-Lerma F, Jorda R, Bermejo B, Catalan Study Group of Nosocomial Pneumonia Prevention: Prevention of nosocomial infection in mechanically ventilated patients: selective digestive decontamination versus sucralfate. Clin Intens Care. 1997;8:228-235

S260. Quinio B, Albanese J, Bues-Charbit M, Viviand X, Martin C; Selective decontamination of the digestive tract in multiple trauma patients. A prospective double-blind, randomized, placebo-controlled study. Chest 1996;109:765-772

S261. Rimola A, Bory F, Teres J, Perez-Ayuso RM, Arroyo V, Rodes J. Oral, nonabsorbable antibiotics prevent infection in cirrhotics with gastrointestinal hemorrhage. *Hepatol*. 1985;5(3):463-7.

S262. Rocha LA, Martin MJ, Pita S, Paz J, Seco C, Margusino L, Villanueva R, Duran MT: Prevention of nosocomial infection in critically ill patients by selective decontamination of the digestive tract. A randomized, double blind, placebo-controlled study. Intensive Care Med. 1992;18:398-404

S263. Rodríguez-Roldán JM, Altuna-Cuesta A, López A, Carrillo A, Garcia J, León J, Martínez-Pellús AJ: Prevention of nosocomial lung infection in ventilated patients: use of an antimicrobial pharyngeal nonabsorbable paste. Crit Care Med. 1990;18:1239-42

S264. Rolando N, Gimson A, Wade J, Philpott-Howard J, Casewell M, Williams R: Prospective controlled trial of selective parenteral and enteral antimicrobial regimen in fulminant liver failure. Hepatol. 1993;17:196-201

S265. Rolando N, Wade JJ, Stangou A, Gimson AE, Wendon J, Philpott-Howard J, Williams R. Prospective study comparing the efficacy of prophylactic parenteral antimicrobials, with or without enteral decontamination, in patients with acute liver failure. Liver Transplant Surgery. 1996; 2:8-13

S266. Sanchez-Garcia M, Cambronero JA, Lopez-Diaz J, et al. Effectiveness and cost of selective decontamination of the digestive tract in critically ill intubated patients. A randomized, double-blind, placebo-controlled multicenter trial. *Am Rev Respir Dis* 1998; 158:908-16.

S267. Sirvent JM, Torres A, El-Ebiary M, Castro P, de Batlle J, Bonet A. Protective effect of intravenously administered cefuroxime against nosocomial pneumonia in patients with structural coma. *Am J Respir Crit Care Med* 1997;155:1729-1734

S268. Smith SD, Jackson RJ, Hannakan CJ, Wadowsky RM, Tzakis AG, Rowe MI; Selective decontamination in pediatric liver transplants. Transplantation 1993;55:1306-1308

S269. Stoutenbeek CP, van Saene HKF, Little RA, Whitehead A: The effect of selective decontamination of the digestive tract on mortality in multiple trauma patients: a multicenter randomized controlled trial. Intensive Care Med. 2007;33:261-270

S270. Ulrich C, Harinck-deWeerd JE, Bakker NC, et al Selective decontamination of the digestive tract with norfloxacin in the prevention of ICU-acquired infections: A prospective randomized study. Intensive Care Med 1989;15:424-431.

S271. Unertl K, Ruckdeschel G, Selbmann HK, et al; Prevention of colonization and respiratory infections in long-term ventilated patients by local antimicrobial prophylaxis. Intensive Care Med 1987;13:106-113

S272. Van Delden C, Köhler T, Brunner-Ferber F, François B, Carlet J, Pechère JC. Azithromycin to prevent Pseudomonas aeruginosa ventilator-associated pneumonia by inhibition of quorum sensing: a randomized controlled trial. Intensive Care Med. 2012 Jul 1;38(7):1118-25.

S273. Verwaest C, Verhaegen J, Ferdinande P, Schetz M, Van den Berghe G, Verbist L, Lauwers P: Randomized, controlled trial of selective digestive decontamination in 600 mechanically ventilated patients in a multidisciplinary intensive care unit. Crit Care Med. 1997;25:63-71

S274. Wiener J, Itokazu G, Nathan C, Kabins SA, Weinstein RA: A randomized, double-blind, placebo-controlled trial of selective digestive decontamination in a medical-surgical intensive care unit. Clin Infect Dis. 1995;20:861-867

S275. Zobel GE, Kuttnig MA, Grubbauer HM, Semmelrock HJ, Thiel WE. Reduction of colonization and infection rate during pediatric intensive care by selective decontamination of the digestive tract. Crit Care Med. 1991;19(10):1242-6

S276. Ables AZ, Blumer NA, Valainis GT, Godenick MT, Kajdasz DK, Palesch YY. Fluconazole Prophylaxis of Severe Candida Infection in Trauma and Postsurgical Patients: A Prospective, Double-Blind, Randomized, Placebo-Controlled Trial. Infect Dis Clin Practice 2000;9(4):169-175.

S277. Eggimann P, Francioli P, Bille J, Schneider R, Wu MM, Chapuis G, Chiolero R, Pannatier A, Schilling J, Geroulanos S, et al (1999) Fluconazole prophylaxis prevents intra-abdominal candidiasis in high-risk surgical patients. Crit Care Med 27:1066–1072.

S278. Garbino J, Lew DP, Romand JA, Hugonnet S, Auckenthaler R, Pittet D: Prevention of severe Candida infections in nonneutropenic, high-risk, critically ill patients: a randomized, double-blind, placebo-controlled trial in patients treated by selective digestive decontamination. Intensive Care Med 2002;28:1708-1717

S279. Giglio M, Caggiano G, Dalfino L, Brienza N, Alicino I, Sgobio A, Favale A, Puntillo F. Oral nystatin prophylaxis in surgical/trauma ICU patients: a randomised clinical trial. Crit Care 2012;16(2):R57.

S280. Jacobs S, Price Evans DA, Tariq M, Al Omar NF (2003) Fluconazole improves survival in septic shock: a randomized double-blind prospective study. Crit Care Med 31:1938–1946.

S281. Lumbreras C, Cuervas-Mons V, Jara P, Del Palacio A, Turrion VS, Barrios C, Paya C V (1996) Randomized trial of fluconazole versus nystatin for the prophylaxis of *Candida* infection following liver transplantation. J Infect Dis 174(3); 583-588.

S282. Normand S, François B, Dardé ML, Bouteille B, Bonnivard M, Preux PM, Gastinne H, Vignon P. Oral nystatin prophylaxis of *Candida* spp. colonization in ventilated critically ill patients. Intensive Care Med 2005;31:1508–1513.

S283. Ostrosky-Zeichner L, Shoham S, Vazquez J, Reboli A, Betts R, Barron MA, Pappas P G. MSG-01: a randomized, double-blind, placebo-controlled trial of caspofungin prophylaxis followed by pre-emptive therapy for invasive candidiasis in high-risk adults in the critical care setting. Clin Infect Dis 2014;58(9):1219-1226.

S284. Parizkova R, Cerny V, Dostal P, Truhlar A (2000) The effect of prophylactic fluconazole administration on fungal infection in critically ill patients. Anesteziologie a neodkladna pece 11:271-5.

S285. Pelz RK, Hendrix CW, Swoboda SM, Diener-West M, Merz WG, Hammond J, Lipsett PA. Double-blind placebo-controlled trial of fluconazole to prevent candidal infections in critically ill surgical patients. Annal Surg. 2001;233(4):542.

S286. Piarroux R, Grenouillet F, Balvay P, Tran V, Blasco G, Millon L, Boillot A Assessment of pre-emptive treatment to prevent severe candidiasis in critically ill surgical patients. Crit Care Med 2004;32(12):2443-2449.

S287. Savino JA, Agarwal N, Wry P, Policastro A, Cerabona T, Austria L. Routine prophylactic antifungal agents (clotrimazole, ketoconazole, and nystatin) in nontransplant nonburned critically ill surgical and trauma patients. J Trauma 1994;36:20–26.

S288. Schuster MG, Edwards JE, Sobel JD, et al (2008) Empirical fluconazole versus placebo for intensive care unit patients: a randomized trial. Ann Intern Med 149:83-90.

S289. Verhaegen J: Randomized study of selective digestive decontamination on colonization and prevention of infection in mechanically ventilated patients in the ICU. 1992. Doctor in Medical Sciences – thesis, University Hospital, Leuven, Belgium.

S290. Klompas M, Speck K, Howell MD, *et al*. Reappraisal of routine oral care with chlorhexidine gluconate for patients receiving mechanical ventilation: systematic review and meta-analysis. *JAMA Intern Med.* 2014; **174**: 751-61.

S291. Zhao T, Wu X, Zhang Q, Li C, Worthington HV, Hua F. Oral hygiene care for critically ill patients to prevent ventilator-associated pneumonia. *Cochrane Database Syst Rev*. 2020(12).

S292. Silvestri L, Weir I, Gregori D, Taylor D, Van Saene J, Van Saene H. Effectiveness of oral chlorhexidine on nosocomial pneumonia, causative microorganisms and mortality in critically ill patients: a systematic review and meta-analysis. Minerva Anestesiol. 2014;80(7):805-20.

S293. Silvestri L, Weir WI, Gregori D, Taylor N, Zandstra DF, van Saene JJ, van Saene HK. Impact of oral chlorhexidine on bloodstream infection in critically ill patients: systematic review and meta-analysis of randomized controlled trials. J Cardiothoracic Vasc Anesth. 2017;31(6):2236-44.

S294. Hurley JC: Prophylaxis with enteral antibiotics in ventilated patients: Selective decontamination or selective cross-infection? *Antimicrob Agents Chemother* 1995; **39**: 941–7.

S295. Minozzi S, Pifferi S, Brazzi L, Pecoraro V, Montrucchio G, D'Amico R. Topical antibiotic prophylaxis to reduce respiratory tract infections and mortality in adults receiving mechanical ventilation. Cochrane Database Syst Rev 2021, Issue 1. Art. No.: CD000022. DOI: 10.1002/14651858.CD000022.pub4.

S296. Silvestri L, Van Saene HK, Milanese M, Gregori D, Gullo A. Selective decontamination of the digestive tract reduces bacterial bloodstream infection and mortality in critically ill patients. Systematic review of randomized, controlled trials. J Hosp Infect. 2007;65(3):187-203.

S297. van Till JO, van Ruler O, Lamme B, Weber RJ, Reitsma JB, Boermeester MA. Single-drug therapy or selective decontamination of the digestive tract as antifungal prophylaxis in critically ill patients: a systematic review. Critical Care. 2007;11(6):1-6.

S298. Silvestri L, Van Saene HK, Milanese M, Gregori D. Impact of selective decontamination of the digestive tract on fungal carriage and infection: systematic review of randomized controlled trials. *Intensive Care Med* 2005, 31:898-910.

S299. Cortegiani A, Russotto V, Maggiore A, Attanasio M, Naro AR, Raineri SM, Giarratano A. Antifungal agents for preventing fungal infections in non-neutropenic critically ill patients. Cochrane Database of Systematic Reviews. 2016(1).

S300. Playford EG, Webster AC, Sorrell TC, Craig JC. Antifungal agents for preventing fungal infections in non-neutropenic critically ill and surgical patients: systematic review and meta-analysis of randomized clinical trials. *J Antimicrob Chemother* 2006 ;57(4):628-38.

Figure S1. (Page 41) Search method, screening criteria and resulting classification of eligible studies and subsequent decant of component groups. The six steps are as follows;
(1) An electronic search for systematic reviews or meta-analysis (SR/MA) containing potentially eligible studies using search terms; "ventilator associated pneumonia", "mechanical ventilation", "intensive care unit", each combined with either "meta-analysis" or "systematic review" up to November 2021; (2) The systematic reviews were then searched for studies of patient populations requiring prolonged (> 24 hours) ICU admission
(3) The studies were triaged from the systematic reviews into one of five categories; studies in which there was no intervention (observational studies), studies of various non-decontamination methods such as methods delivered either via the gastric route, the airway route or via the oral care route, studies of anti-septic methods, studies of antibiotic-based interventions, and studies of single drug antifungal (SAF) prophylaxis.
(4) All studies were reviewed for potentially eligible studies and screened against inclusion and exclusion criteria. Any duplicate or ineligible studies were removed and
(5) Studies identified outside of systematic reviews were included;
(6) The component groups were decanted from each study being control (rectangles), intervention (ovals) and observation (diamond) groups.
Note; the total numbers do not tally as some systematic reviews provide studies in more than one category and some studies provide groups in more than one category and some studies have unequal numbers of control and interventions groups. TAP = Topical antibiotic prophylaxis

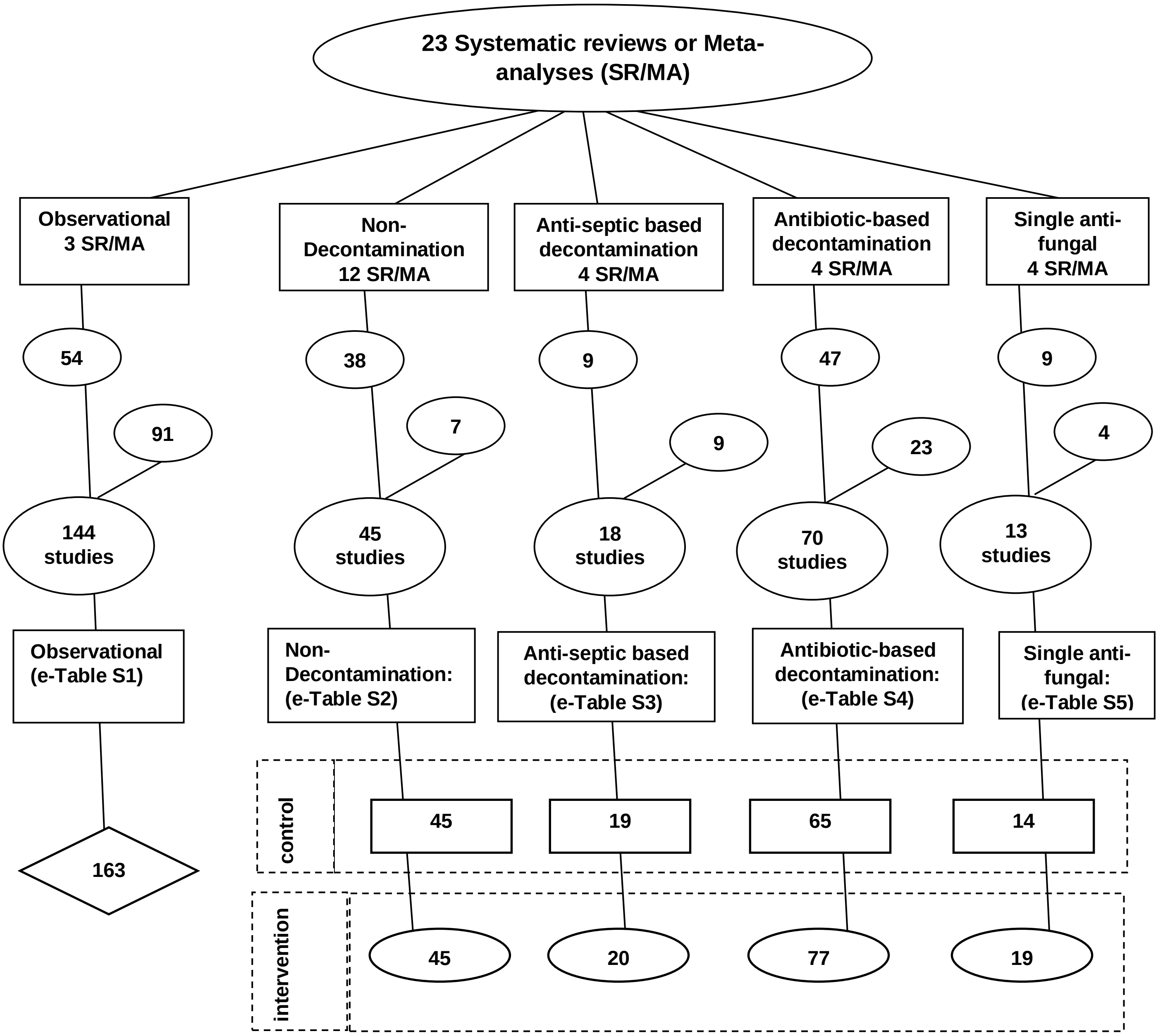
23 Systematic reviews or Meta-analyses (SR/MA)
Observational 3 SR/MA
Non-Decontamination 12 SR/MA
Anti-septic based decontamination 4 SR/MA
Antibiotic-based decontamination 4 SR/MA
Single anti-fungal 4 SR/MA
54
38
9
47
9
91
7
9
23
4
144 studies
45 studies
18 studies
70 studies
13 studies
Observational (e-Table S1)
Non-Decontamination: (e-Table S2)
Anti-septic based decontamination: (e-Table S3)
Antibiotic-based decontamination: (e-Table S4)
Single anti-fungal: (e-Table S5)
control
45
19
65
14
163
intervention
45
20
77
19

Figure s2 Effect size *Staph aureus bacteremia*

S aureus bacteremia

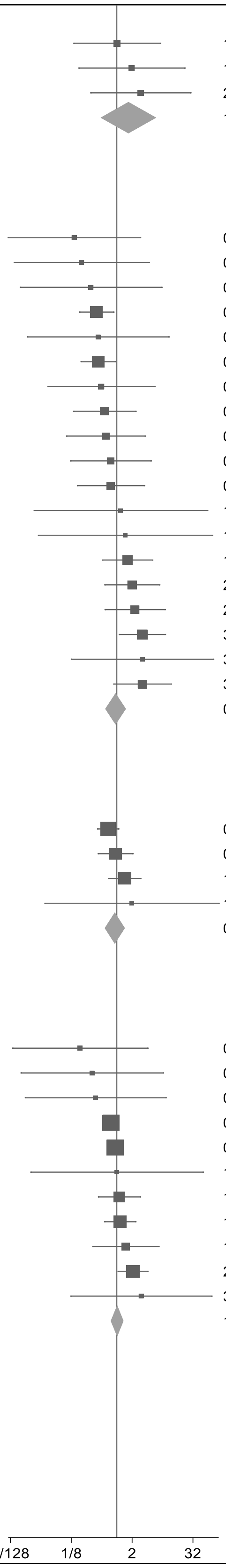

Study	Treatment Yes	Treatment No	Control Yes	Control No	Odds ratio with 95% CI	Weight (%)
**nil**						
Holzapfel_I_99	2	197	2	198	1.01 [0.14, 7.21]	1.25
Camus '05 PT&MCh	2	127	1	125	1.97 [0.18, 21.99]	0.86
Camus '05 PT	3	127	1	125	2.95 [0.30, 28.77]	0.96
Heterogeneity: τ^2 = 0.00, I^2 = 0.00%, H^2 = 1.00					1.69 [0.48, 6.00]	
Test of $\theta_i = \theta_j$: Q(2) = 0.51, p = 0.77						
Test of θ = 0: z = 0.81, p = 0.42						
**amphotericin ± tap**						
Rolando '93	0	28	3	28	0.14 [0.01, 2.89]	0.57
Ferrer	0	39	2	39	0.20 [0.01, 4.30]	0.55
Stoutenbeek'84 SDD	0	63	1	58	0.31 [0.01, 7.69]	0.50
de Smet NC SDD	9	2,036	22	1,968	0.40 [0.18, 0.86]	5.24
Rolando	0	47	1	60	0.42 [0.02, 10.66]	0.50
de Smet NC SOD	9	1,895	22	1,968	0.42 [0.20, 0.93]	5.24
Gaussorgues	1	58	2	57	0.49 [0.04, 5.57]	0.85
Stoutenbeek'87 ED	3	39	7	52	0.57 [0.14, 2.35]	2.22
Camus '14	2	1,020	3	922	0.60 [0.10, 3.61]	1.48
Rocha	2	45	3	51	0.76 [0.12, 4.73]	1.42
Hartenauer	3	96	4	97	0.76 [0.17, 3.48]	1.96
Jacobs	0	36	0	43	1.19 [0.02, 61.56]	0.34
Bion	0	21	0	31	1.47 [0.03, 76.71]	0.33
Stoutenbeek '07	8	193	5	195	1.62 [0.52, 5.03]	3.15
Quinio	8	68	4	68	2.00 [0.58, 6.96]	2.73
Verwaest OA	7	186	3	182	2.28 [0.58, 8.97]	2.35
de la Cal	14	39	6	53	3.17 [1.12, 8.99]	3.57
Laggner	1	32	0	34	3.18 [0.13, 81.01]	0.49
Verwaest PTA	10	190	3	182	3.19 [0.86, 11.79]	2.53
Heterogeneity: τ^2 = 0.35, I^2 = 37.78%, H^2 = 1.61					0.94 [0.59, 1.51]	
Test of $\theta_i = \theta_j$: Q(18) = 26.03, p = 0.10						
Test of θ = 0: z = -0.25, p = 0.80						
**Nystatin ± tap**						
SuDDICU	27	2,764	46	3,145	0.67 [0.41, 1.08]	8.28
Wittekamp PTNy	12	2,212	13	2,238	0.93 [0.43, 2.05]	5.17
Wittekamp PTNy	17	2,065	13	2,238	1.42 [0.69, 2.93]	5.68
Gorensek	0	17	0	34	1.97 [0.04, 103.61]	0.33
Heterogeneity: τ^2 = 0.06, I^2 = 26.69%, H^2 = 1.36					0.91 [0.57, 1.44]	
Test of $\theta_i = \theta_j$: Q(3) = 3.11, p = 0.37						
Test of θ = 0: z = -0.41, p = 0.68						
**chlorhexidine**						
Cabov	0	30	2	28	0.19 [0.01, 4.06]	0.54
Fourrier'00	0	30	1	29	0.32 [0.01, 8.24]	0.49
Bleasdale	0	391	1	444	0.38 [0.02, 9.32]	0.50
Huang_3UD	92	25,932	80	17,276	0.77 [0.57, 1.03]	10.51
Huang_2TD	106	24,646	70	15,148	0.93 [0.69, 1.26]	10.48
Swan	0	161	0	161	1.00 [0.02, 50.70]	0.34
Climo_CRT	9	1,401	8	1,390	1.12 [0.43, 2.90]	4.03
Noto_post	16	4,472	15	4,837	1.15 [0.57, 2.34]	5.85
Milstone	3	664	4	1,322	1.49 [0.33, 6.69]	2.02
Wittekamp Chlx	25	2,083	13	2,238	2.07 [1.05, 4.05]	6.15
Fourrier'05	1	113	0	114	3.03 [0.12, 75.08]	0.50
Heterogeneity: τ^2 = 0.05, I^2 = 27.09%, H^2 = 1.37					1.01 [0.76, 1.35]	
Test of $\theta_i = \theta_j$: Q(10) = 10.16, p = 0.43						
Test of θ = 0: z = 0.08, p = 0.94						
Heterogeneity: τ^2 = 0.11, I^2 = 31.76%, H^2 = 1.47						
Test of $\theta_i = \theta_j$: Q(36) = 40.92, p = 0.26						
Test of θ = 0: z = -0.08, p = 0.93						
Test of group differences: Q_b(3) = 0.88, p = 0.83						

Random-effects REML model
Sorted by: _meta_es

Figure s3 Effect size *Candidemia*

Candidemia

Study	Treatment Yes	Treatment No	Control Yes	Control No	Peto's OR with 95% CI	Weight (%)
**nil**						
Holzapfel_I_99 1999	1	198	1	199	1.01 [0.06, 16.12]	1.08
Heterogeneity: τ^2 = 0.00, I^2 = .%, H^2 = .					1.01 [0.06, 16.12]	
Test of $\theta_i = \theta_j$: Q(0) = -0.00, p = .						
Test of θ = 0: z = 0.00, p = 1.00						
**amphotericin ± tap**						
Godard 1990	0	97	2	82	0.11 [0.01, 1.86]	1.08
Verwaest PTA 1997	0	200	4	181	0.12 [0.02, 0.88]	1.91
Stoutenbeek'84 SDD 1984	0	63	1	58	0.13 [0.00, 6.39]	0.58
Verwaest OA 1997	0	193	4	181	0.13 [0.02, 0.91]	1.91
Smith 1993	0	18	1	17	0.14 [0.00, 6.82]	0.58
Zobel 1991	0	25	1	24	0.14 [0.00, 6.82]	0.58
Winter 1992	0	91	1	91	0.14 [0.00, 6.90]	0.58
Rolando 1996	0	47	1	60	0.17 [0.00, 8.87]	0.57
Gaussorgues 1991	5	54	15	44	0.30 [0.12, 0.79]	4.61
Camus '14 2014	1	1,021	2	923	0.46 [0.05, 4.47]	1.52
de Smet NC SDD 2009	8	2,037	16	1,974	0.50 [0.22, 1.11]	5.31
de Smet NC SOD 2009	14	1,890	16	1,974	0.91 [0.45, 1.88]	5.71
Stoutenbeek '07 2007	1	200	0	200	7.35 [0.15, 370.54]	0.58
Blair 1991	1	125	0	130	7.63 [0.15, 384.58]	0.58
Hartenauer 1990	3	96	0	101	7.70 [0.79, 74.85]	1.52
de la Cal 2005	1	52	0	59	8.27 [0.16, 419.38]	0.58
Heterogeneity: τ^2 = 0.29, I^2 = 26.92%, H^2 = 1.37					0.49 [0.27, 0.87]	
Test of $\theta_i = \theta_j$: Q(15) = 21.77, p = 0.11						
Test of θ = 0: z = -2.42, p = 0.02						
**Nystatin ± tap**						
Wiener 1995	0	30	2	29	0.14 [0.01, 2.21]	1.07
Cerra 1992	1	24	4	17	0.22 [0.03, 1.38]	2.11
Gorensek 1993	0	17	1	33	0.22 [0.00, 14.26]	0.52
Cockerill 1992	1	74	2	73	0.51 [0.05, 4.97]	1.51
Wittekamp PTNy 2018	18	2,064	15	2,236	1.30 [0.65, 2.58]	5.87
Wittekamp PTNy 2018	23	2,201	15	2,236	1.55 [0.82, 2.93]	6.11
Savino_Ny 1994	5	38	2	37	2.28 [0.49, 10.63]	2.71
Heterogeneity: τ^2 = 0.00, I^2 = 0.00%, H^2 = 1.00					1.20 [0.79, 1.83]	
Test of $\theta_i = \theta_j$: Q(6) = 8.11, p = 0.23						
Test of θ = 0: z = 0.87, p = 0.39						
**azoles & other ± tap**						
Eggimann 1999	0	23	2	18	0.11 [0.01, 1.84]	1.06
Schuster 2008	0	122	2	125	0.14 [0.01, 2.25]	1.08
Piarroux 2004	0	428	6	449	0.14 [0.03, 0.71]	2.57
Jacobs 2003	0	32	1	38	0.16 [0.00, 8.32]	0.58
Ostrosky-Zeichner 2014	1	116	7	95	0.18 [0.04, 0.75]	3.04
Garbino_Fl 2002	1	102	9	92	0.18 [0.05, 0.65]	3.46
Savino_Cl 1994	1	54	2	37	0.35 [0.03, 3.53]	1.47
Pelz 2001	1	129	3	127	0.36 [0.05, 2.61]	1.90
Savino_Ket 1994	1	27	2	37	0.70 [0.07, 7.18]	1.46
Heterogeneity: τ^2 = 0.00, I^2 = 0.00%, H^2 = 1.00					0.21 [0.11, 0.40]	
Test of $\theta_i = \theta_j$: Q(8) = 2.11, p = 0.98						
Test of θ = 0: z = -4.80, p = 0.00						
**chlorhexidine**						
Noto_post 2015	2	4,486	6	4,846	0.40 [0.10, 1.59]	3.11
Climo_CRT 2013	7	1,403	16	1,382	0.45 [0.20, 1.02]	5.22
Huang_2TD 2013	63	24,689	56	15,162	0.68 [0.47, 0.99]	7.37
Huang_3UD 2013	62	25,962	59	17,297	0.69 [0.48, 1.00]	7.40
Milstone 2013	3	664	6	1,320	0.99 [0.25, 3.98]	3.11
Bleasdale 2007	2	389	2	443	1.14 [0.16, 8.15]	1.91
Wittekamp Chlx 2018	22	2,086	15	2,236	1.57 [0.82, 2.99]	6.06
Heterogeneity: τ^2 = 0.05, I^2 = 32.33%, H^2 = 1.48					0.75 [0.55, 1.03]	
Test of $\theta_i = \theta_j$: Q(6) = 7.99, p = 0.24						
Test of θ = 0: z = -1.75, p = 0.08						
Heterogeneity: τ^2 = 0.30, I^2 = 47.61%, H^2 = 1.91						
Test of $\theta_i = \theta_j$: Q(39) = 62.17, p = 0.01						
Test of θ = 0: z = -3.54, p = 0.00						
Test of group differences: $Q_b(4)$ = 21.83, p = 0.00						

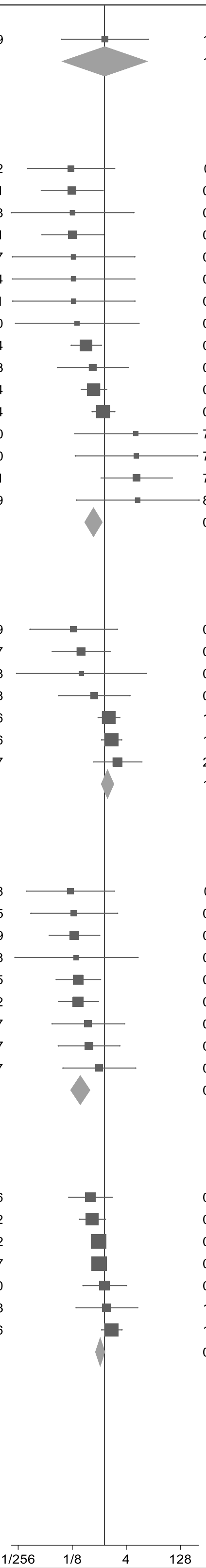

Random-effects REML model
Sorted by: _meta_es

Figure s4. **GSEM of the interaction model (Model C) in relation to *Staphylococcus* and *Candida* infection data.** *Candida* col and *Staphylococcus* col (ovals) are latent variables representing *Candida* and *Staphylococcus* colonization, respectively. The variables in rectangles are binary predictor variables representing the group level exposure to the following; patient selection for candidemia risk factors (CRF); trauma ICU setting (trauma50), mean or median length of ICU stay ≥ 7 days (los7), exposure to a topical anti-septic (a_S), exposure to TAP (tap), concurrency of a control group with an antibiotic-based intervention group (CC), exposure to a non-decontamination based prevention method (non-D), greater than 90% use of mechanical ventilation (mvp90) or exposure to PPAP (ppap). Note that the model factorizes exposures from compound regimens (e.g. SDD and SOD, which combine TAP, an antifungal together with or without PPAP) into singleton TAP, PPAP and anti-fungal exposures. The circles contain error terms. The three part boxes represent the binomial data for *Candida* and *Staphylococcus* VAP (v_can_n, v_sr_n) and candidemia (b_can_n) or bacteremia (b_sr_n) counts with the number of patients as the denominator which is logit transformed using the logit link function in the generalized model.

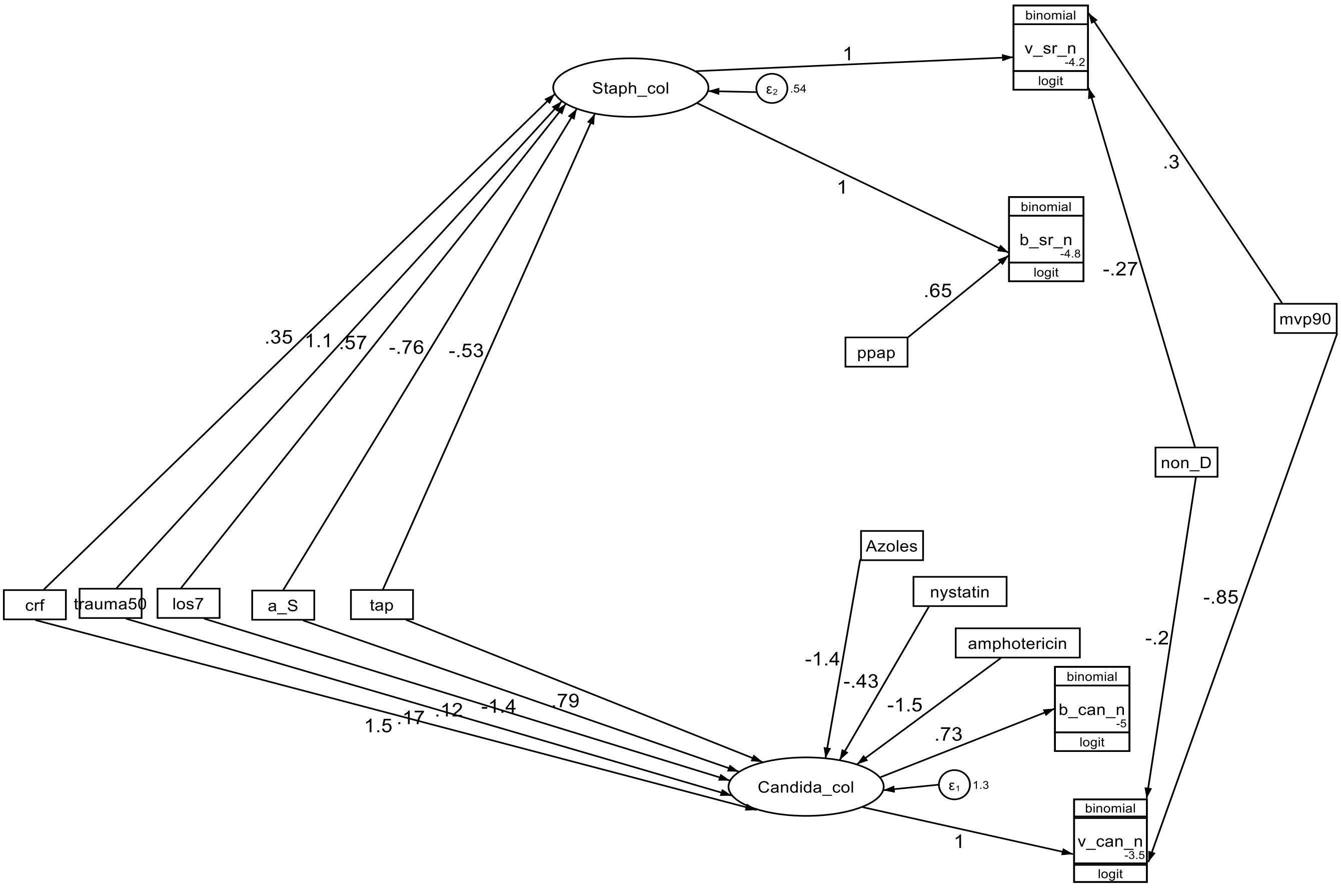

Figure s5. **GSEM of the interaction model (Model B) in relation to *Staphylococcus* and *Candida* infection data.** *Candida* col and *Staphylococcus* col (ovals) are latent variables representing *Candida* and *Staphylococcus* colonization, respectively. The variables in rectangles are binary predictor variables representing the group level exposure to the following; patient selection for candidemia risk factors (CRF); trauma ICU setting (trauma50), mean or median length of ICU stay ≥ 7 days (los7), exposure to a topical anti-septic (a_S), exposure to TAP (tap), concurrency of a control group with an antibiotic-based intervention group (CC), exposure to a non-decontamination based prevention method (non-D), greater than 90% use of mechanical ventilation (mvp90) or exposure to PPAP (ppap). Note that the model factorizes exposures from compound regimens (e.g. SDD and SOD, which combine TAP, an antifungal together with or without PPAP) into singleton TAP, PPAP and anti-fungal exposures. The circles contain error terms. The three part boxes represent the binomial data for *Candida* and *Staphylococcus* VAP (v_can_n, v_sr_n) and candidemia (b_can_n) or bacteremia (b_sr_n) counts with the number of patients as the denominator which is logit transformed using the logit link function in the generalized model.

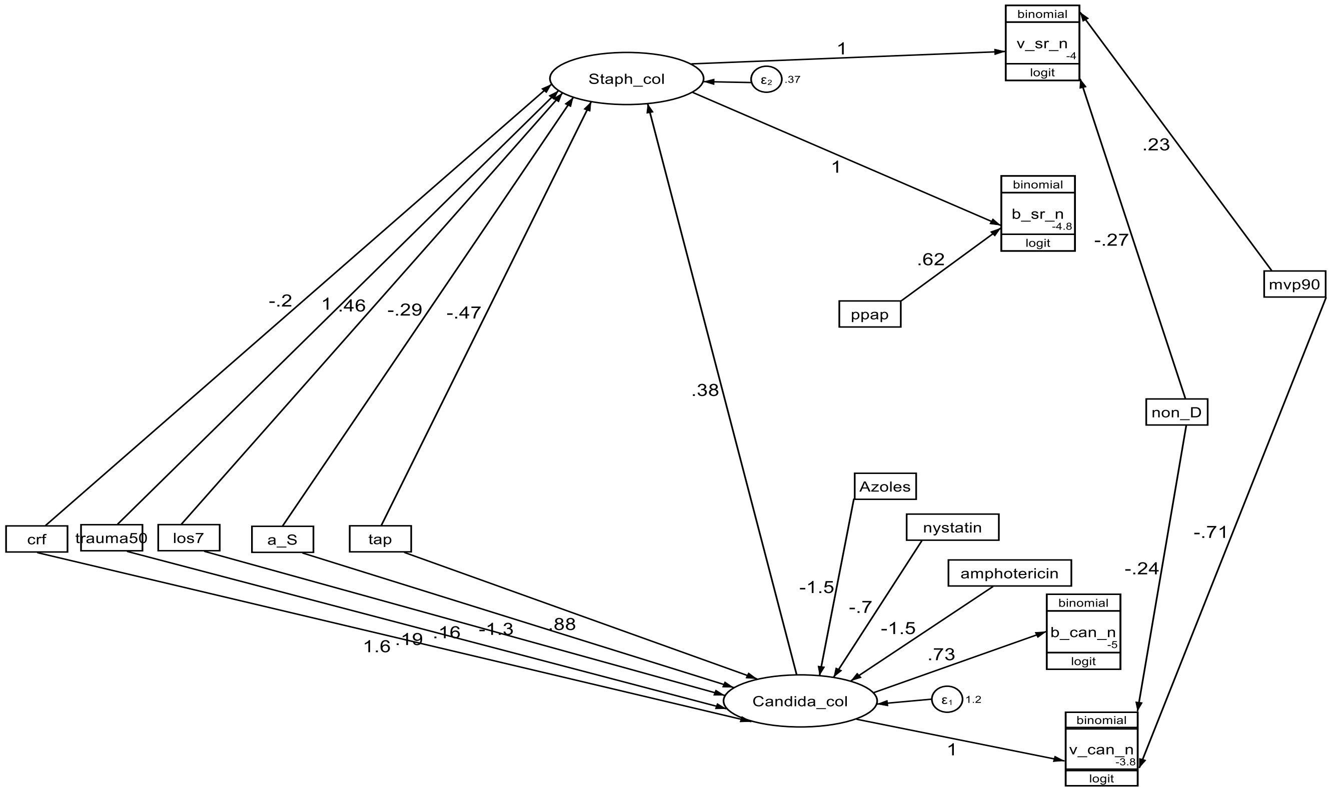

Figure s6. Model A projections for *Staphylococcus aureus* bacteremia (fig S8a) and Candidemia (Fig S8b) incidences among groups of patients from non-trauma ICU groups with mean LOS>7 days and not selected for candidemia risk factors (CRF). The benchmark incidence in each plot is the summary mean derived from the Non-concurrent control (NCC) projected mean (central vertical line). The projections are for control (top) or intervention (bottom panel) groups receiving prophylaxis with various singleton or combination interventions. non-D is non-decontamination, A_s is anti-septic, TAP is topical antibiotic

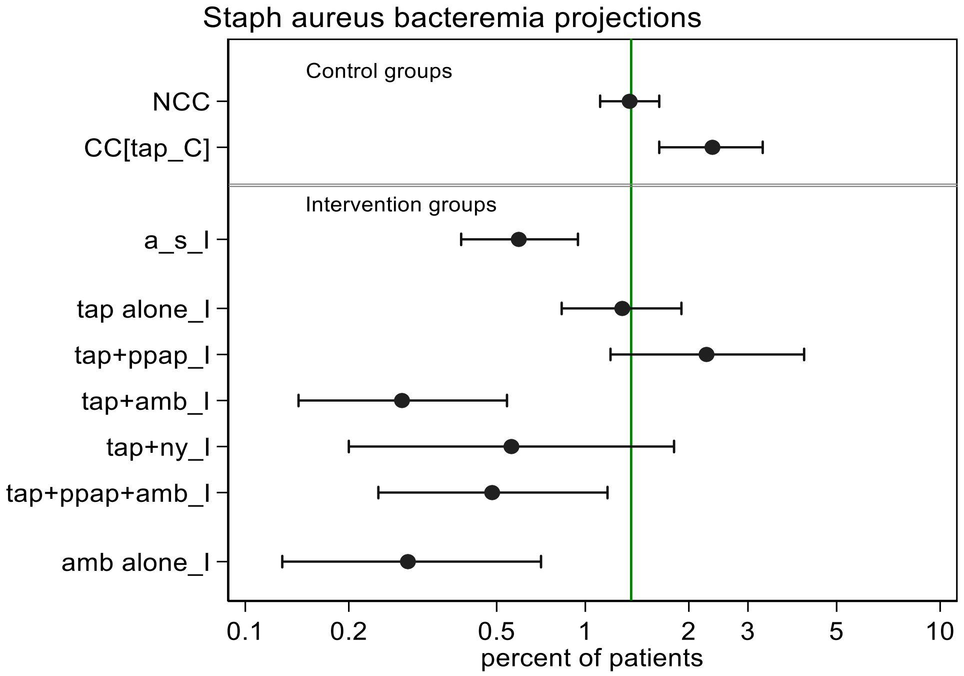

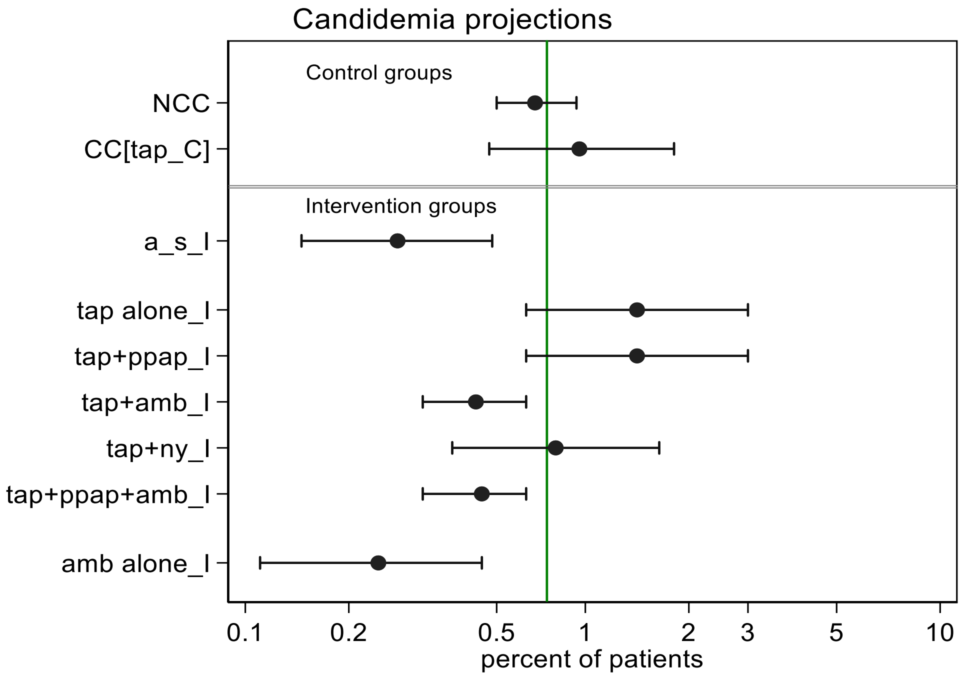

Supplement: Supplementary file 1 — Supplementary file1 (PDF 2453 KB) [file 10096_2023_4573_MOESM1_ESM.pdf]
